# Selection and Gene Duplication Associated With High-Elevation Diversification in *Pristimantis*, the Largest Terrestrial Vertebrate Genus

**DOI:** 10.1093/gbe/evae167

**Published:** 2024-08-07

**Authors:** Nicholas Christodoulides, Veronica L Urgiles, Juan M Guayasamin, Anna E Savage

**Affiliations:** Department of Biology, University of Central Florida, Orlando, FL, USA; Department of Biology, University of Central Florida, Orlando, FL, USA; Departamento de herpetologia, Instituto Nacional de Biodiversidad del Ecuador, Quito, Ecuador; Colegio de Ciencias Biológicas y Ambientales COCIBA, Instituto Biósfera, Laboratorio de Biología Evolutiva, Universidad San Francisco de Quito USFQ, Quito, Ecuador; Ingeniería en Biodiversidad y Recursos Genéticos, Centro de Biodiversidad y Cambio Climático BioCamb, Universidad Tecnológica Indoamérica, Quito, Ecuador; Department of Biology, University of Central Florida, Orlando, FL, USA

**Keywords:** transcriptomics, *Pristimantis*, direct development, adaptation, anurans, frogs

## Abstract

The genus *Pristimantis* diversified in the tropical Andes mountains and is the most speciose genus of terrestrial vertebrates. *Pristimantis* are notable among frogs in that they thrive at high elevations (>2,000 m) and are direct developers without a tadpole stage. Despite their ecological significance, little is known about the genetic and physiological traits enabling their success. We conducted transcriptomic analysis on seven *Pristimantis* species sampled across elevations in the Ecuadorean Andes to explore three hypotheses for their success: (i) unique genes are under selection relative to all other frogs, (ii) common selection occurs across all direct developers, or (iii) common selection occurs across all high-elevation frog clades. Comparative analysis with 34 frog species revealed unique positive selection in *Pristimantis* genes related to aerobic respiration, hemostasis, signaling, cellular transportation of proteins and ions, and immunity. Additionally, we detected positive selection across all direct developers for genes associated with oxygenase activity and metal ion binding. While many genes under selection in *Pristimantis* were not positively selected in other high-elevation frog species, we identified some shared genes and pathways linked to lipid metabolism, innate immunity, and cellular redox processes. We observed more positive selection in duplicated- versus single-copy genes, while relaxed purifying selection was prevalent in single-copy genes. Notably, copy number of an innate immunity complement gene was positively correlated with *Pristimantis* species elevation. Our findings contribute novel insights into the genetic basis of adaptation in *Pristimantis* and provide a foundation for future studies on the evolutionary mechanisms leading to direct development and coping with high elevations.

**Significance**

The genus *Pristimantis* is a hyper-diverse clade of frogs that develop terrestrially and can live at extreme elevations in the Andes Mountain range. Despite their dominance among Andean amphibians, the molecular evolution of *Pristimantis* frogs remains poorly understood and no formal comparative genomics analyses have been undertaken thus far. In this study, we compared new transcriptome sequences of seven *Pristimantis* species to those of other frog species and tested for positive selection and duplication in candidate genes that may underlie *Pristimantis* diversification, evolution of direct development, and adaptation to high-elevation environments. We find many genes uniquely under positive selection in *Pristimantis* that contribute to our understanding of how this clade diversified into high-elevation ecosystems. We also find numerous genes and pathways selected for across high-elevation species and direct developers that provide targets for functional analysis in future studies of these life history traits.

## Introduction

How organisms adapt to variable, novel, or extreme environments and how their genomes reflect advantageous traits are important questions in evolutionary biology (Hendry 2016; Wellborn and Langerhans 2015). Identifying the molecular drivers of adaptation, especially in speciose taxa that seem to have rapidly diversified across different habitats, can help to establish a framework for studying how changes to organisms’ genes relate to their evolutionary trajectories (e.g. Schneider et al. 2019 and Torres-Dowdall et al. 2015). The tropical Andes of South America is the world's most biodiverse hotspot (Hoorn et al. 2010). Andean amphibians are particularly diverse, especially frogs of the genus *Pristimantis*, which comprise the most species-rich monophyletic complex of terrestrial vertebrates currently known (Frost 2014). *Pristimantis* are small, direct-developing frogs that lack a tadpole stage, often live at high elevations (>3,000 m) and are entirely terrestrial (Hedges et al. 2008). Although numerous studies have described and delineated cryptic species within *Pristimantis* using morphology and DNA barcoding (Rivera-Correa et al. 2017; Urgiles et al. 2019), the ecological, physiological, or genetic attributes underlying their prolific evolutionary success at high elevations remain unclear.

The Andes region's complex topography and elevational gradients likely influence the extraordinary diversification of anurans (Hazzi et al. 2018). When a species or group of species expands over heterogeneous landscapes, differing selective pressures from the environment drive genetic variation within and between diverging populations (Barrett and Schluter 2008). In some cases, these selection disparities can spur adaptive diversification, or the proliferation of specializations and sub-lineages as they adapt to environments they encounter (Wellborn and Langerhans 2015). Until recently, selection associated with adaptive diversification has typically been studied through the lens of phenotypic evolution, incorporating relatively few genomic data features (Sella and Barton 2019). Studies focusing on selection across the genome level are becoming more prevalent in species-rich taxa known to originate from rapid diversification events (Marques et al. 2018; Schneider et al. 2019), but phylogenomic and transcriptomic approaches have not yet been applied to *Pristimantis* lineages.

Evolutionary diversification can be facilitated by novel environments, but access to these environments may also become available due to unique features of the taxa which go on to occupy them. Species complexes which have undergone recent speciation often share a key innovation—a trait or set of traits that relax constraints of adaptive evolution on ecologically important traits and allow lineages to transition and exploit ecological opportunities in different ways (Maia et al. 2013). For example, the ability of *Pristimantis* to inhabit so much ecological space across elevational gradients may be related to specialized genes or gene expression which allow them to cope with low oxygen levels in the high-elevation Andes. As another option, direct development may have had a more significant role in the genomic diversification of *Pristimantis* than their high-elevation environments. Direct development has repeatedly evolved as a reproductive mode in frogs from ancestors that bred entirely in water and is associated with features such as reduced clutch size, larger egg size, reduced adult size, parental care, and occurrence in wetter and warmer regions (Gomez-Mestre et al. 2012). Little has been published on genomic features potentially related to these traits, nevertheless, substantial alterations to life history traits have been linked to higher levels of diversification in other anurans such as New World frogs that exclusively breed in plants that accumulate water (Tonini et al. 2020).

Expansion of important gene families via duplication may also be an important factor in driving diversification in *Pristimantis*. Both orthologs (gene copies that arise via speciation events) and paralogs (arising through duplication events) can enable evolutionary diversification by relaxation of purifying selection on at least one gene copy, which eventually either loses its function and becomes a pseudogene or gains a novel function (i.e. neofunctionalization) (Sandve et al. 2018). Gene copies arising from gene duplication events are usually more divergent from their ancestral variant in both nucleotide sequence and expression levels, and thus have a higher chance of neofunctionalization (Fares et al. 2013). Previous work has found evidence that gene families with higher rates of duplication are more likely to experience positive or relaxed selection in diverse taxa that underwent rapid diversification and subsequent specialization such as aphids (Boulain et al. 2018) and multiple fish lineages (Daane and Deitrich 2022; Malinsky et al. 2018). If similar evolutionary dynamics take place in *Pristimantis*, we can also expect duplicated genes to be more likely to be under relaxed purifying selection and increased positive diversifying selection compared to single-copy ones. Alternately, if the ancestral gene function is not lost, having multiple copies of a gene to express in response to variable conditions can be an advantageous starting point for a generalist flexible ancestor to produce more specialized descendant lineages (Malinsky et al. 2018). Even without subsequent diversification or specialization of gene copies, multiple gene copies can translate to more gene product and higher gene dosage, which could impact fitness in environments that require higher levels of transcriptional output such as high elevations (Kondrashov 2012).

Phenotypic and genomic diversification commonly result from geographic barriers such as mountains preventing gene flow between isolated populations. Small changes in environmental conditions that span gradations, like elevation, can promote further disparity by imposing differential selective pressures on pre-existing genetic variation within the populations (Wellborn and Langerhans 2015). We can glean information about the types and strength of positive selection which occurred in *Pristimantis* by aligning their gene sequences to orthologous sequences of other frog species. Complete frog genomes are still lacking for most lineages, primarily due to the large size and high complexity of their genomes creating assembly challenges (Womack et al. 2022). However, RNA sequencing (RNAseq) does not present the same limitation, and transcriptomic datasets are readily available across numerous frog lineages (Schott et al. 2022). Transcriptome resources can be used to examine which types of selection are acting at the level of expressed gene sequences, provided that orthology is considered properly (Cheon et al. 2020). Comparing other frog transcript sequences relative to *Pristimantis* in a phylogenetic context offers an opportunity to pinpoint genes that are evolving under positive selection or have undergone expansion and whether these evolutionary changes are unique to *Pristimantis* or are found in other direct developers and high-elevation species.

In this study, we characterized gene duplication and selection in *Pristimantis* and other frogs to understand phenotypic adaptations to high-elevation environments and the evolutionary transition to direct development. Specifically, we evaluate three hypotheses for the success of *Pristimantis* based on transcriptomic data: (i) *Pristimantis* has unique genes that were selected for life at high elevations, (ii) all direct developers show the same genes under selection, or (iii) all high-elevation frog species show the same genes under selection. We used a phylotranscriptomic pipeline with rigorous quality control to analyze newly generated *Pristimantis* transcriptomes and existing data across other frog species for signals of positive selection. We tested whether paralogs duplicated within *Pristimantis*, direct developers, or high-elevation species are more likely to undergo relaxed versus intensified selective pressure than single-copy ones and summarized candidate gene groups that show significant changes among species tested in each hypothesis. We also used a phylogenetic mixed modeling approach to test whether variation in duplicated orthogroup copy numbers corresponds to the elevational ranges occupied by included frog species. Our study contributes to recent efforts to sequence anuran transcriptomes and illustrates the value of using transcriptome data in comparative phylogenetic and evolutionary studies.

## Results

### Novel *Pristimantis* Transcriptomes

Sequencing generated an average of 693,447 paired end reads per transcriptome library (range: 501,900 to 860,846). The seven *Pristimantis* assemblies totaled 56,618 transcripts with an N50 of 537 bp (supplementary table S1, Supplementary Material online). Of these, 28,533 transcripts had at least one blast result in the Uniref90 database. Overall, *Pristimantis* assemblies were missing large percentages of metazoan BUSCOs (supplementary fig. S2, Supplementary Material online).

### Single-Copy Orthogroups and Species Tree Construction

**Fig. 1.**
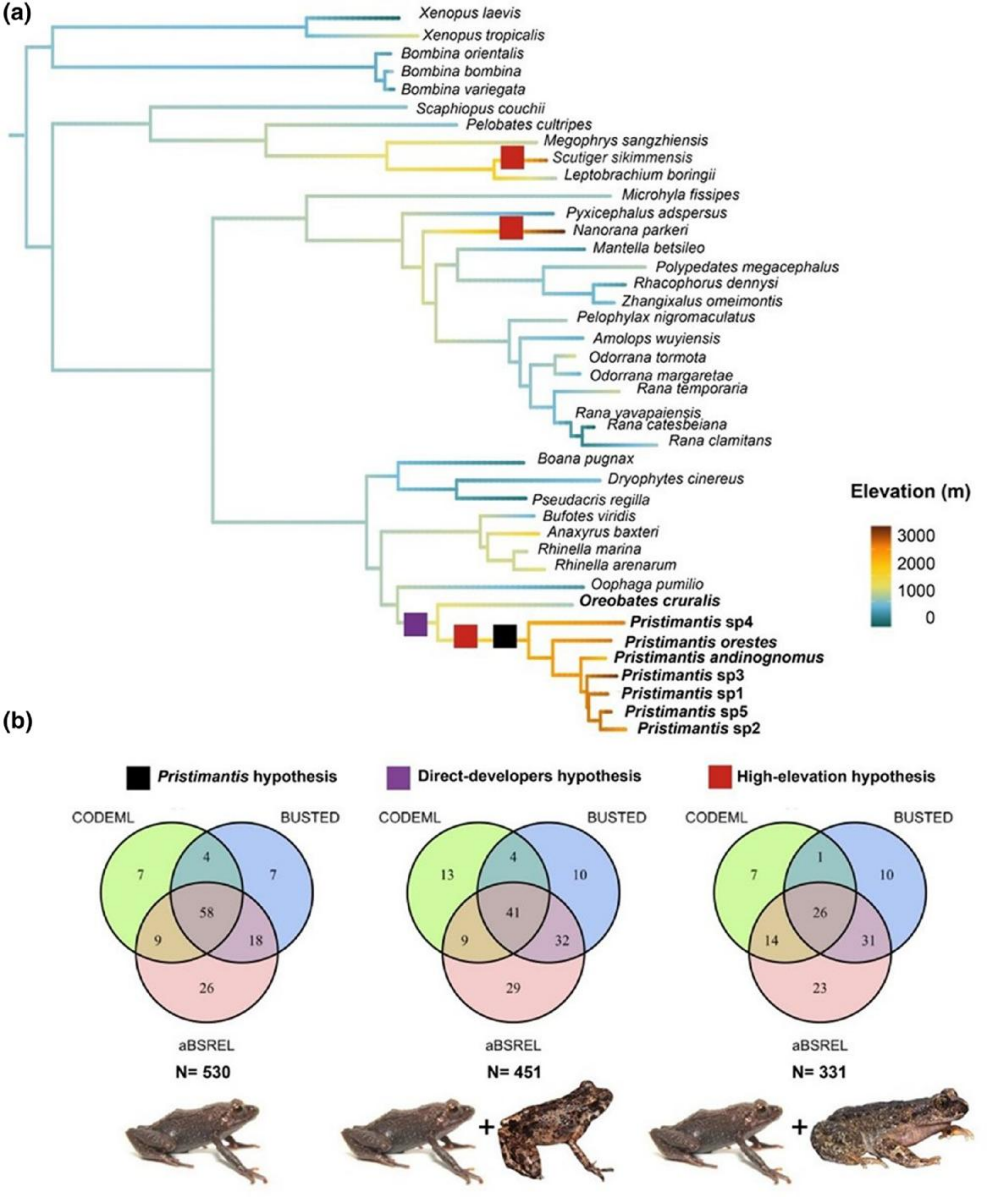
a) Species tree generated from running IQTree on concatenated alignment of all orthogroups retained by PhyloPyPruner. This tree is a midpoint-rooted representation of the phylogeny used for analysis (supplementary fig. S7, Supplementary Material online), shown here for ease of visualization. All species tree nodes have bootstrap support values > 95%. Species included as foreground to test our three evolutionary hypotheses are shown to the right in shades of green (Hypothesis 1: *Pristimantis*-specific selection, Hypothesis 2: selection across direct developers, Hypothesis 3: selection across high-elevation species). These species and nodes were also the ones analyzed for significant evidence of orthogroup duplication. The elevation values for all species are reconstructed across the branches in the phylogeny using FastANC. b) Venn diagrams representing the number of single-copy orthogroups with significant evidence of positive selection detected across three methods and their overlap among the three evolutionary hypotheses. *N* refers to the total number of orthogroups tested for each hypothesis.

Across all frog transcriptomes, 876,261 coding sequences (85.2% of total) were assigned to 48,831 orthogroups, 32,066 of which yielded at least one annotation from InterproScan. Only 66 orthogroups contained sequences from all 41 species, and none of them were single-copy orthologs. We found 2,829 annotated orthogroups containing at least one *Pristimantis* sequence, of which 9 were found only in *Pristimantis* species (supplementary table S2, Supplementary Material online). Removal of paralogous sequences with PhyloPyPruner resulted in a final dataset of 530 unique gene alignments of single-copy orthologs containing at least one *Pristimantis* species sequence. The species tree topology generated from these concatenated single-copy alignments was consistent with previously described anuran phylogenies (Fig. 1a) (Feng et al. 2017). Removal of alignments missing the direct developer *Oreobates cruralis*, and alignments missing either of the non-*Pristimantis* high-elevation species (*Nanorana parkeri* or *Scutiger sikkimensis*), resulted in 451 and 331 orthogroups available to test the direct developer and high-elevation foreground hypotheses, respectively.

### Signals of Positive Selection in *Pristimantis*, Direct Developers, and High-Elevation Species

From the 530 tested single-copy orthogroup alignments, BUSTED detected significant gene-wide positive selection in 87 orthogroups when only *Pristimantis* species were designated as foreground branches. BUSTED also found 87 and 68 positively selected orthogroups with all direct developers and high-elevation species set as foreground, respectively (adj. *P* < 0.05; Fig. 1b). The average alignment-wide *ω* value was significantly higher in foreground species than background species when *Pristimantis* was set as the foreground (paired *t*-test, *P* = 3.75e^−12^), as well as for direct developers (*P* = 1e^−04^), but not for high-elevation species (*P* = 0.13; supplementary fig. S3, Supplementary Material online). The aBSREL analysis found 111 orthogroups with significant evidence of positive diversifying selection in *Pristimantis* only, 111 orthogroups in all direct developers, and 94 orthogroups in high-elevation species (Fig. 1b). These orthogroups had significantly elevated *ω* rate categories in foreground branches in a small proportion of sites (2.22 to 4.67% for *Pristimantis*, 2.10 to 5.04% for direct developers, and 1.64 to 5.51% for high-elevation species; supplementary tables S5 to S7, Supplementary Material online). Finally, CODEML analysis found 78 orthogroups under positive selection within *Pristimantis*, 67 orthogroups in direct developers, and 48 orthogroups in high-elevation species (Fig. 1b). For all three hypotheses, these orthogroups yielded significantly higher *ω* values in positively selected foreground branches compared to in background branches (paired *t*-test, *Pristimantis P* = 2.2e^−16^, direct developer *P* = 8.062e^−14^, high-elevation *P* = 2.2e^−16^).

**Fig. 2.**
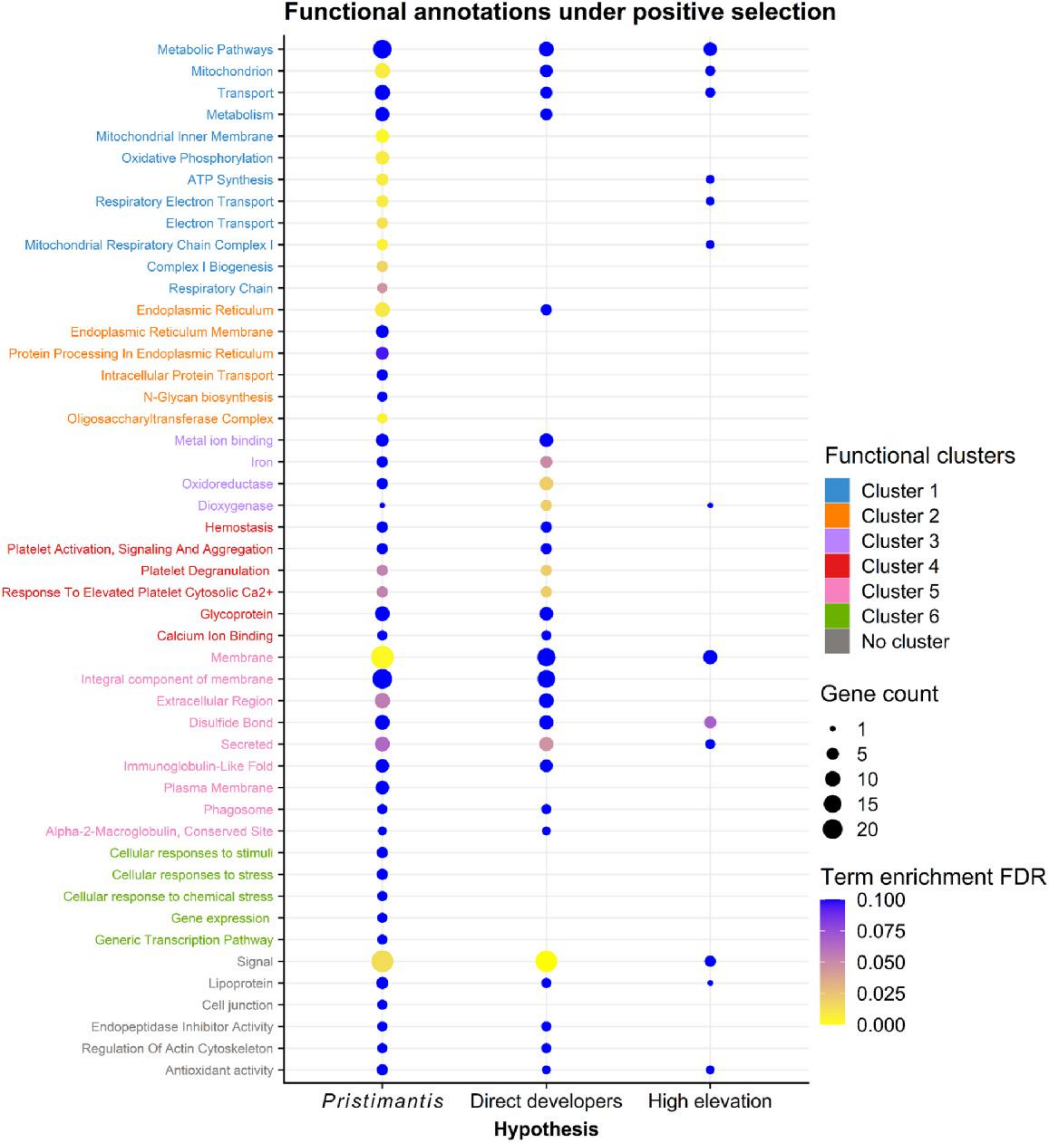
Enrichment scores of annotation terms across the positively selected orthogroups (including both duplicated- and single-copy) identified for each of the three evolutionary hypotheses (*Pristimantis* only, direct developers, and high-elevation species). The identified GO, KEGG, and Reactome Pathway terms were obtained using DAVID and grouped with terms that share similar annotations with the DAVID Functional Annotation Clustering function. Term groupings represent enriched annotation clusters, which were assigned number labels in the analysis results report. Dot color indicates the FDR-corrected *P*-values of the DAVID hypergeometric test for term enrichment, with yellow colors indicating higher significance. Dot size is proportional to the number of identified positively selected orthogroups.

We found significant evidence of positive selection in all three tests (BUSTED, aBSREL, and CODEML) for 58 orthogroups in *Pristimantis*, 41 in direct developers, and 26 in high-elevation species (Fig. 1b, supplementary tables S5 to S7, Supplementary Material online). Single-copy orthogroups under positive selection in the *Pristimantis* hypothesis were clustered into functional groups characterized by pathways for intracellular protein transport and modification within the endoplasmic reticulum, membrane composition and signaling, stress response, and mitochondrial respiration and metabolism, including multiple subunits of the mitochondrial NADH dehydrogenase (ubiquinone) complex (Fig. 2). When we tested for positive selection in the direct developer hypothesis, we identified some significant genes that did not appear when testing the *Pristimantis* hypothesis, including genes involved in nucleic acid binding (TOP1MT, DDX21, HIRIP3, DKC1, EWSR1), redox homeostasis and mitochondrial function (APOOL, QSOX, MRPS33, MRPL58), dioxygenase activity and metal ion binding (BCO2, ARL6IP4, ETHE1, SDHC), and fatty acid metabolism (APOB, ACBD5) (supplementary table S4, Supplementary Material online). Lastly for the high-elevation species hypothesis, we found positive selection in membrane composition and metabolism genes and genes involved in the formation of protein disulfide bonds (Fig. 2). We found four orthogroups (HSD11B1, ANXA5, GNAI2, PCBD2) that were under positive selection in the high-elevation hypothesis but not the *Pristimantis* hypothesis (supplementary table S4, Supplementary Material online).

### Functions of Single-Copy Genes Under Relaxed and Intensified Selection

**Fig. 3.**
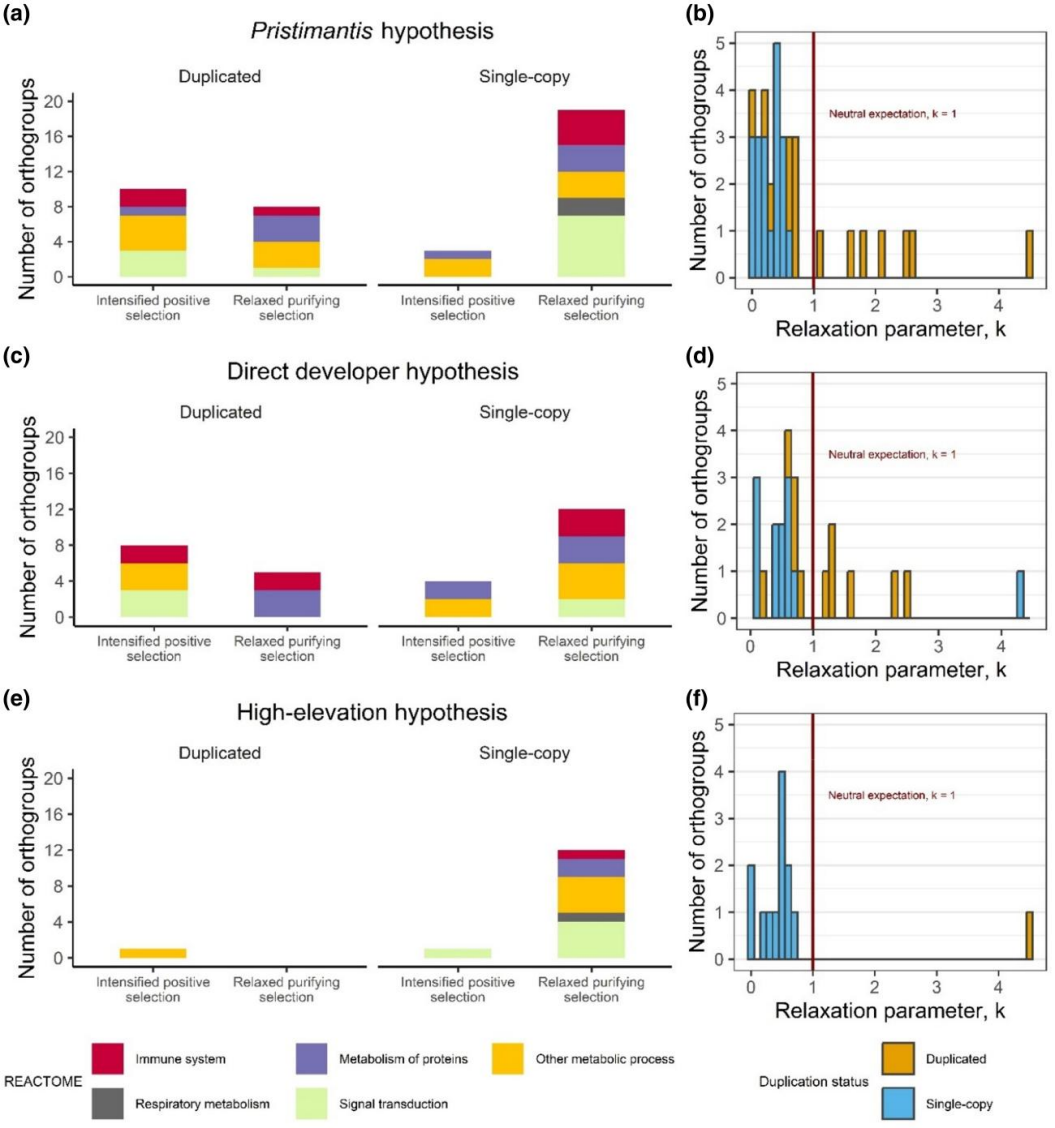
Distribution of single-copy and duplicated orthogroups with significant RELAX results for the three evolutionary hypotheses: (a, b) *Pristimantis* only, (c, d) direct developers, and (e, f) high-elevation species tested as foreground. Bar height corresponds to the number of orthogroups under significant relaxed purifying or intensified positive selection (left), and the histogram plots display the distribution of *k*-values among intensified or relaxed orthogroups in contrast with the neutral expectation of 1 (right). Bar colors represent parent molecular gene pathway classifications based on analysis results from the Reactome pathway knowledgebase (left) and duplication status of significant orthogroups (right).

We ran RELAX on orthogroups with significant results across all three positive selection tests to determine whether observed increases in nucleotide substitution rates were a result of relaxed purifying (relaxation parameter *k* < 1) or intensified positive selection (*k* > 1). In *Pristimantis*, RELAX identified 19 orthogroups with significant evidence of relaxed purifying selection (*k* < 1, *P* < 0.05), and 3 orthogroups with intensified positive selection (*k* > 1, *P* < 0.05) (Fig. 3a). In direct developers, RELAX identified 12 orthogroups with significant evidence of relaxed purifying selection and 4 orthogroups with intensified positive selection (Fig. 3c). Across high-elevation species, RELAX identified 12 orthogroups with evidence of relaxed purifying selection and one orthogroup with intensified positive selection (Fig. 3e). In *Pristimantis*, the median relaxation parameter *k* was 0.694 across all orthogroups and differed significantly from the neutral expectation of 1 (Wilcoxon signed-rank test: *V* = 448, *P* = 0.002). Relaxation of selection happened significantly more often than intensification of selection among orthogroups in *Pristimantis* (Fisher's Exact Test, *P* = 2e^−04^) (Fig. 3a and b). In direct developers, average *k* did not significantly differ from the expected null of 1 (*V* = 311, *P* = 0.124), but relaxation of selection was more prevalent than intensification (*P* = 0.048) (Fig. 3c and d). In high-elevation species, the median relaxation parameter *k* was 0.662 across all orthogroups and was significantly lower than the neutral expectation of 1 (Wilcoxon signed-rank test: *V* = 60, *P* = 0.002), and relaxation of selection happened significantly more often than intensification of selection among orthogroups (*P* = 8e^−04^) (Fig. 3e and f).

We found eight single-copy orthogroups under relaxed purifying selection exclusively in the *Pristimantis* hypothesis. These included genes with functions in mitochondrial electron transport (COX4I1, NDUFA2), cell junction formation (OCLN, NECTIN2), phosphatase activity (STYX, ADTRP), and regulation of cell proliferation and differentiation (ATRAID, CREG1) (supplementary table S4, Supplementary Material online). Additionally, we found three single-copy orthogroups that showed significant evidence of relaxation for at least two of our three hypotheses (*Pristimantis*, direct developers, and/or high-elevation species): MBL2 and C8G, which are involved in complement activation pathways, and TMEM205, whose function is not well characterized but was identified as a candidate gene under selection in the highland American pika (Ge et al. 2013). MBL2 was significantly relaxed for all three hypotheses, C8G was under relaxed selection in *Pristimantis* and direct developers, and TMEM205 was under relaxed selection in *Pristimantis* and high-elevation species. The 40S ribosomal protein fragment A (RPSA) was the only gene with evidence for intensified positive selection exclusively for the *Pristimantis* hypothesis, and sulfotransferase (SULT1C1) and apolipoprotein A1 (APOA1) had stronger intensified positive selection in *Pristimantis* compared to direct developers (supplementary tables S5 to S7, Supplementary Material online).

Six single-copy orthogroups were under relaxed purifying selection exclusively in direct developers: hydroxysteroid 11-beta-dehydrogenase 1 (HSD11B1), retbindin (Rtbdn), mitochondrial ribosomal subunit S33 (MRPS33), organic solute transporter subunit beta (SLC51B), coxsackievirus and adenovirus receptor (CXADR), and an uncharacterized oxidoreductase. Orthogroups with strongest support for relaxed selection in direct developers were apolipoprotein M (APOM), which was under relaxed purifying selection all three hypotheses, and a calcium-binding EF-hand protein (EFHD1), which was significant in *Pristimantis* and direct developers (supplementary table S4, Supplementary Material online).

Exclusively in the high-elevation species hypothesis, we found six orthogroups under relaxed purifying selection: annexin V (ANXA5), nicotinamide-N methyltransferase (NNMT), NADH-ubiquinone oxidoreductase beta subcomplex subunit 4 (NDUFB4), junctional adhesion molecule A (F11R), p53 and DNA damage-regulated protein 1 (PDRG1), and biogenesis of lysosome-related organelles complex 1 (BLOC1). Guanine nucleotide-binding protein subunit alpha-2 (GNAI2) showed intensified positive selection exclusively in high-elevation species (Table 3). In both *Pristimantis* and high-elevation species, we found relaxed purifying selection in hydroxysteroid 17-beta dehydrogenase 13 (HSD17B13), a ribosome biogenesis protein (SLX9), and RWD domain-containing protein 4 (RWDD4), but there was stronger relaxation of selection in high-elevation species.

### Selection in Duplicated Orthogroups

STRIDE detected 77 unique orthogroups with significant evidence of a gene duplication event (≥0.5 STRIDE support value) in nodes within the *Pristimantis* clade. Only one orthogroup, annotated as a cytochrome P450, also showed evidence of a duplication event at the node grouping *Pristimantis* with the other direct developer, and this orthogroup was duplicated again within the *Pristimantis* clade. Of these 77 orthogroups, 75 were assigned an annotation by either DIAMOND or InterproScan, comprising our final dataset for selection and phylogenetic generalized least-squares (PGLS) analyses (supplementary tables S8 to S10, Supplementary Material online). Duplicated orthogroups were overrepresented for Gene Ontology (GO) terms including endopeptidase inhibitor activity (false discovery rates [FDR] = 0.009), extracellular region (FDR = 0.04), and extracellular space (FDR = 0.04) relative to all orthogroups with an annotation. Duplicated orthogroups were also enriched for macroglobulin-like coding domains associated with these GO terms (FDR = 0.001); supplementary fig. S5, Supplementary Material online). Seven of these orthogroups were duplicated in *Pristimantis* and in the other two high-elevation frog species (*N. parkeri* and *S. sikkimensis*). Two orthogroups are cytochrome P450s involved in mitochondrial respiration (CYP2G1, CYP2B6), while the others have functions in cell adhesion and wound healing (FN1), proteinase inhibition (A2ML1), hydrolase activity (uncharacterized carboxylic ester hydrolase), RNA binding (HNRNPA3), and regulation of redox reactions (NMRAL1).

**Fig. 4.**
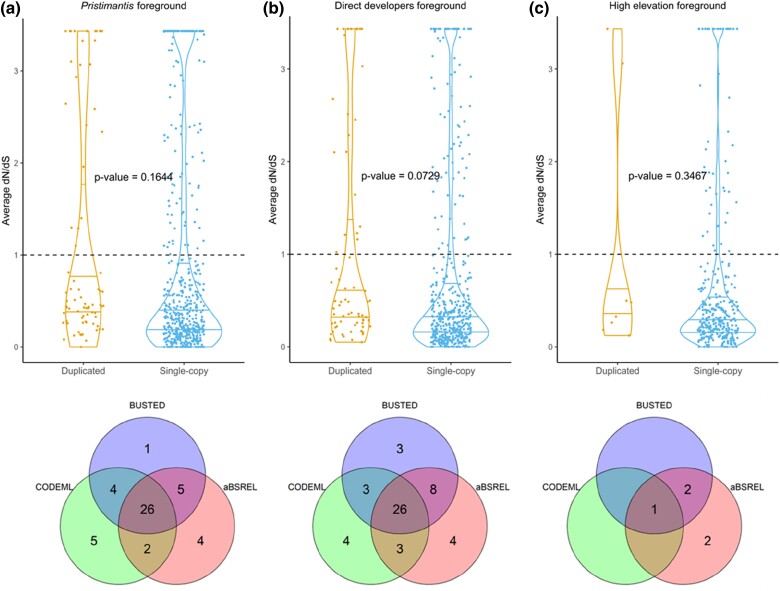
Violin plot of average dN/dS (*ω*) values estimated by BUSTED for duplicated- versus single-copy orthogroups with a) *Pristimantis*, b) direct developers, and c) high-elevation species set as foreground branches and all other species set as background species. *P*-values are from two-way unpaired *t*-tests comparing average foreground *ω* values between duplicated- and single-copy orthogroups for each hypothesis. The Venn diagrams under each plot show overlap among hypotheses in the orthogroups with significant evidence of positive selection across statistical tests, with counts of orthogroups in each category.

Significant positive selection was detected in 26 of the 75 tested duplicated orthogroups for both the *Pristimantis* and direct developer hypotheses across all three tests (Fig. 4a and b). Only one of the high-elevation duplicated orthogroups, redox sensor gene NMRAL1, was significant in the three tests (Fig. 4c). Duplicated orthogroups were significantly more likely to show signals of positive selection across all three tests compared to single-copy orthogroups for *Pristimantis* (Fisher's Exact Test, *P* = 8.72e^−07^) and direct developers (*P* = 7.257e^−08^), but not for high-elevation species (*P* = 0.4889). However, BUSTED foreground *ω* was not significantly different from background *ω* in duplicated genes tested for *Pristimantis*, direct developers, or high-elevation species (paired *t*-test). Additionally, the average alignment-wide *ω* values did not significantly differ between duplicated- and single-copy orthogroups for any of the three foreground hypotheses (unpaired *t*-test; Fig. 4).

RELAX analysis results showed no evidence that duplicated orthogroups were more likely to experience relaxed versus intensified selection for any of our tested hypothesis (Fisher's Exact Tests). Likewise, the average *k* relaxation values for duplicated orthogroups did not significantly differ from the null expectation of 1 (Wilcoxon signed-rank test). However, we found ten duplicated orthogroups with significant evidence of intensified positive selection and eight with relaxed purifying selection in *Pristimantis* (supplementary table S8, Supplementary Material online). Three of these genes were under relaxed purifying selection exclusively in the *Pristimantis* clade. These duplicated genes were dolichyl-diphosphooligosaccharide glycosyltransferase subunit 2 (RPN2), cystathionine-beta synthase (CBS), and flavin-containing monooxygenase (FMO5.2). Lysosome-associated membrane protein 2 (LAMP2), eukaryotic translation initiation factor 3 subunit G (EIF3G) and complement C3 duplicated genes were under relaxed selection in *Pristimantis* and direct developers, with stronger evidence of relaxation in *Pristimantis*. Genes with evidence for intensified positive selection exclusively in *Pristimantis* included aspartate aminotransferase (GOT2), and four genes annotated with biological process GO terms for regulating cellular homeostasis and proliferation: SID1 transmembrane family member 2 (SIDT2), eukaryotic translation initiation factor 4B (EIF4B), dolichyl-diphosphooligosaccharide-protein glycosyltransferase 48 kDa subunit (DDOST), and serine incorporator 3 (SERINC3). The *Pristimantis* hypothesis inferred more significant intensified positive selection in ornithine decarboxylase (ODC1) and hepatitis A virus cellular receptor 1 (HAVCR1) compared to the direct developer hypothesis (supplementary table S4, Supplementary Material online).

For the direct developer hypothesis, RELAX identified five duplicated orthogroups with significant evidence of relaxed purifying selection and eight orthogroups with intensified positive selection. Complement C2 (CFB), sulfhydryl oxidase (QSOX1), and DNA topoisomerase 1 (TOP1MT) genes were under relaxed purifying selection exclusively in the direct developer hypothesis, and acyl-CoA-binding domain-containing protein 5 (ACBD5) was exclusively under intensified positive selection. Peroxisomal thiolase (ACAA1) and calnexin (CANX) were more significant for relaxed selection in direct developers, while fetuin B (FETUB) and fibronectin (FN1) were under more significant intensified selection compared to when tested in *Pristimantis* alone. Lastly, the duplicated NMRAL1 orthogroup was significant for intensified positive selection in both the *Pristimantis* and high-elevation hypotheses but showed slightly stronger intensified selection when tested only in *Pristimantis*.

### Phylogenetic Tests of Relationships Between High-Elevation Dwelling and Orthogroup Duplications

**Fig. 5.**
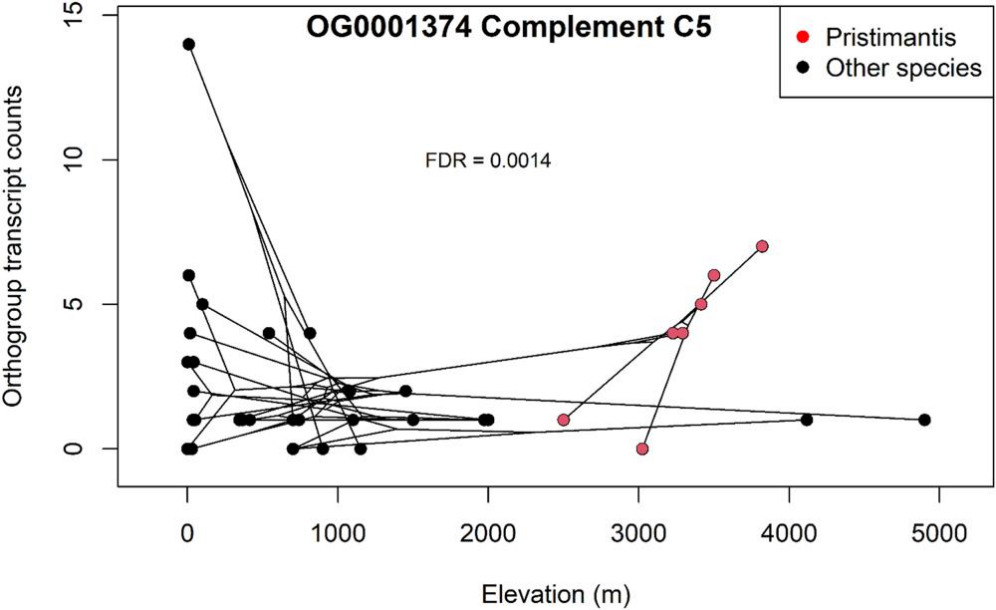
Plot of species elevation versus copy numbers of the Complement C5 (OG0001374) orthogroup with connections between datapoints representing the network of phylogenetic relationships. Dots represent leaves of the phylogenetic tree, and their connections are imposed from the phylogenetic tree used for all analyses. Gene counts show a significant positive correlation with elevation within the *Pristimantis* clade (colored in red; FDR = 0.0014) but not among all species (FDR = 0.988).

Of the 75 annotated orthogroups duplicated within *Pristimantis* or direct developers, six showed significant correlations between the number of ortholog duplications and elevation across all included frog species (raw *P* < 0.05; supplementary table S11, Supplementary Material online). One of these orthogroups, the calcium-binding protein calnexin (CANX), was under relaxed purifying selection in *Pristimantis* (*P* = 0.03) and direct developers (*P* = 0.02). After applying multiple testing correction, however, no correlations were significant (FDR *P* > 0.05). When we tested for the same relationships exclusively within the *Pristimantis* clade, we identified one complement protein orthogroup (OG0001374, complement C5) in which sequence copy number was significantly positively associated with elevation after correction (FDR = 0.001, Fig. 5).

After FDR correction, neither the percentage of selected sites nor the *ω* values (from aBSREL) of duplicated orthogroups were significantly correlated with species elevation, including when examining *Pristimantis* species only. Likewise, we saw no significant relationship between RELAX *k*-values of any of these orthogroups and elevation. When *ω* values in sites under selection were analyzed as principal components for all orthogroups simultaneously, there was no relationship with elevation for any hypothesis in both duplicated- and single-copy orthogroups.

## Discussion

Our study identifies the genes uniquely under selection in *Pristimantis* frogs of the Ecuadorean Andes and contrasts these genes with those commonly selected across direct developers and high-elevation species. We also inferred gene duplication events and find that duplications and positive selection are not generally associated with life at high elevations, with the expection of an innate immunity orthogroup (complement C5) correlated with elevation exclusively in *Pristimantis*. Many of the duplicated- and single-copy genes under positive selection in *Pristimantis* have been previously identified in studies of high-elevation adaptation, and have important functions in immune response, oxidative metabolism, and cell signaling (Tables 1 and 2). The inclusion of the other direct developer, *O. cruralis*, in the tested species was accompanied by positive selection in additional genes involving oxygenase activity and nucleic acid binding. Across all high-elevation species, most genes under selection were also significant when tested only in *Pristimantis*, but we identified three genes involved in similar pathways that were only under selection in the other high-elevation species but not in *Pristimantis*. Intriguingly, we find that duplicated genes are more likely to evolve under positive selection compared to single-copy genes, but show no difference in relaxed purifying selection, suggesting that duplicated gene copies take on new functions without being released from purifying selection.

**Table 1:** The top ten most significant single-copy orthogroups for each hypothesis based on adjusted *P*-values from BUSTED

Hypothesis	Entrez ID	Gene symbol	Adj. BUSTED *P*-value	Hypoxia Adaptation Study DOI	Relevant biochemical pathway or gene function information
*Pristimantis*	100379942	SEPP1	2.06e^−13^	10.1093/gbe/evz101	Selenium transport or responsible for extracellular antioxidant defense properties of selenium
	448083	SULT1C1	6.47e^−13^	10.1126/science.1189406	Sulfonate donor to catalyze the sulfate conjugation of drugs, xenobiotic compounds, hormones, and neurotransmitters
	394919	EFHD1	7.36e^−13^	10.1093/molbev/msab161	Acts as a calcium sensor for mitochondrial flash (mitoflash) activation, an event characterized by stochastic bursts of superoxide production
	100127600	SLX9	7.65e^−13^	…	Ribosome biogenesis factor
	550004	NDUFB10	4.82e^−11^	…	Part of Complex I mediates the transfer of electrons from NADH to the respiratory chain
	100496170	Rtbdn	5.70e^−11^	10.1093/molbev/msy208	Riboflavin-binding protein which might have a role in retinal flavin transport
	100145587	HYPK	9.19e^−11^	10.1371/journal.pgen.1001116	Involved in protein stabilization and folding, negative regulation of apoptotic process
	100135184	RDH16	9.48e^−11^	…	Oxidoreductase acting on retinols, may play part in electron transport chain
	733568	TMEM205	9.02e^−10^	10.1038/ncomms2860	Membrane protein, but very little functional information available
	548364	PDRG1	9.02e^−10^	…	Unfolded protein binding and stabilization
Direct developers	NA	FXR2	6.60e^−09^	10.1093/nsr/nwz213	RNA binding and may regulate intracellular transport and local translation of certain mRNAs
	100134992	IGF2	2.28e^−07^	…	The insulin-like growth factors possess growth-promoting activity. Major fetal growth hormone in mammals. Plays a key role in regulating fetoplacental development
	496828	CLK2	4.26e^−07^	10.1126/science.1189406	Acts as a suppressor of hepatic gluconeogenesis and glucose output by repressing PPARGC1A transcriptional activity on gluconeogenic genes via its phosphorylation
	100145077	DKC1	1.95e^−06^	…	Telomere maintenance
	100497751	PEBP1	3.60e^−06^	…	Serine protease inhibitor which inhibits thrombin, neuropsin, and chymotrypsin but not trypsin, tissue type plasminogen activator, and elastase
	100135124	HIRIP3	4.31e^−06^	…	May play a role in chromatin function and histone metabolism via its interaction with HIRA and histones
	100216278	APOOL	1.29e^−05^	10.1016/j.isci.2018.11.034	Plays a crucial role in crista junction formation and mitochondrial function
	496650	SDHC	1.91e^−05^	…	Part of Complex II of the mitochondrial electron transport chain, related to heme binding, metal binding
	496497	CXADR	0.000201	…	Essential for tight junction integrity. Results in proliferation and production of cytokines and growth factors by T-cells that stimulate epithelial tissues repair
	100487516	SLC51B	0.00130	…	Essential component of the Ost-alpha/Ost-beta complex. Efficiently transports the major species of bile acids. Modulates SLC51A glycosylation, membrane trafficking, and stability activities
High elevation	394861	GNAI2	0	10.1038/ncomms2860	Involved as modulators or transducers in various transmembrane signaling systems. Involved in hormonal regulation of adenylate cyclase. May play a role in cell division
	100496214	MBL2	7.69e^−11^	10.1186/s13059-021-02382-3	Calcium-dependent lectin involved in innate immune defense. Binds to late apoptotic cells, facilitating their uptake by macrophages. May bind DNA
	493545	ANXA5	2.23e^−10^	10.1093/molbev/msab161	This protein is an anticoagulant protein that acts as an indirect inhibitor of the thromboplastin-specific complex, which is involved in the blood coagulation cascade.
	NA	NA	4.89e^−10^	…	Putative oxidoreductase YteT
	100038157	C8G	0.0000229	10.1186/s40246-022-00395-y	C8 is a constituent of the membrane attack complex. The gamma subunit seems to be able to bind retinol.
	496676	NNMT	0.0000041	10.1093/molbev/msv175	Can also N-methylate other pyridines structurally related to nicotinamide and play a role in xenobiotic detoxification
	NA	FXR2	8.19e^−05^	See above	…
	496682	HSD17B13	2.11e_−04_	…	May act by metabolizing steroids, lipids, and retinol
	493384	F11R	0.000408	10.1021/acs.jproteome.8b00911	Contributes to the regulation of the variety of cellular processes, as diverse as epithelial/endothelial barrier function, hemostasis angiogenesis hematopoiesis
	100037868	APOM	0.000638	10.1089/ham.2016.0012	Probably involved in lipid transport. Can bind sphingosine-1-phosphate, myristic acid, palmitic acid and stearic acid, retinol, all-trans-retinoic acid, and 9-cis-retinoic acid

Entrez IDs are based on orthologs from *X. tropicalis*. Orthogroups significant in the direct developer and high-elevation hypotheses were filtered for positive diversifying selection in *O. cruralis* and *N. parkeri/S. sikkimensis*, respectively (aBSREL results). Genes that have been previously identified in studies as candidates in high-altitude adaptation have a DOI provided. Relevant pathways or functional information for positively selected genes were summarized from gene descriptions in iHypoxia database.

**Table 2:** The top five most significant duplicated orthogroups for each hypothesis based on adjusted *P*-values from BUSTED

Hypothesis	Entrez ID	Gene symbol	Adj. BUSTED *P*-value	Hypoxia Adaptation Study DOI	Relevant biochemical pathway or gene function information
*Pristimantis*	100492598	SIDT2	0	10.1093/nsr/nwz213	Key proteins in autophagy-lysosomal degradation pathways
	100491991	HAVCR1	0	10.1126/science.1189406	May play a role in T-helper cell development and the regulation of asthma and allergic diseases
	549716	NMRAL1	0	10.1038/s41598-023-27490-x	Redox sensor protein. Undergoes restructuring and subcellular redistribution in response to changes in intracellular NADPH/NADP(+) levels. Related to the synthesis of nitric oxide and may play a role in the activation of inflammatory processes
	100216255	C3	0	10.1093/nsr/nwz213	C3 plays a central role in the activation of the complement system. Its processing by C3 convertase is the central reaction in both classical and alternative complement pathways
	100145597	DDOST	0	…	Required for efficient N-glycosylation. Subunit of the oligosaccharyl transferase (OST) complex that catalyzes the initial transfer of a defined glycan to nascent polypeptide chains, the first step in protein N-glycosylation
Direct developers	496992	FETUB	0	…	Protease inhibitor required for egg fertilization. Required to prevent premature zona pellucida hardening before fertilization by inhibiting the protease activity of ASTL, a protease that mediates the cleavage of ZP2 and triggers zona pellucida hardening
	100127718	PRDX5	7.84e^−13^	10.1371/journal.pone.0093314	Thiol-specific peroxidase catalyzes the reduction of hydrogen peroxide and organic hydroperoxides to water and alcohols, respectively. Plays a role in cell protection against oxidative stress by detoxifying peroxides and as sensor of hydrogen peroxide-mediated signaling events
	595087	FN1	2.39e^−12^	…	Fibronectins bind cell surfaces and various compounds, involved in cell adhesion, cell motility, opsonization, wound healing, and maintenance of cell shape healing, and maintenance of cell shape (by similarity). Essential for osteoblast mineralization
	734085	DDX21	0.000174	…	Binds various RNAs, and promotes rRNA transcription, processing, and modification (by similarity). Required for rRNA 2′-O-methylation, possibly by promoting the recruitment of late-acting snoRNAs with pre-ribosomal complexes
	100497751	ERGIC2	0.000114	10.1093/molbev/msy208	Possible role in transport between endoplasmic reticulum and Golgi
High elevation	549716	NMRAL1	3.81e^−10^	See above	…

Entrez IDs are based on orthologs from *X. tropicalis*. Orthogroups significant in the direct developer and high-elevation hypotheses were filtered for positive diversifying selection in *O. cruralis* and *N. parkeri/S. sikkimensis*, respectively (aBSREL results). Genes that have been previously identified in studies as candidates in high-altitude adaptation have a DOI provided. Relevant pathways or functional information for positively selected genes were summarized from gene descriptions in iHypoxia database.

**Table 3:** Top five orthogroups under relaxed or intensified selection for each hypothesis based on *P*-values from RELAX analysis

Hypothesis(es)	Entrez ID	Gene symbol	Status	RELAX *P*-value	RELAX *k*-Value	Relevant biochemical pathway or gene function information
*Orthogroups under relaxed purifying selection*						
Elevation	493545	ANXA5	Single-copy	8.70e^−10^	0.3182	Calcium ion binding, negative regulation of coagulation
Direct developers	100487516	SLC51B	Single-copy	6.81e^−06^	0.0940	Essential component of the Ost-alpha/Ost-beta complex. Efficiently transports the major species of bile acids. Modulates SLC51A glycosylation, membrane trafficking, and stability activities
*Pristimantis* Direct developers	100038157	C8G	Single-copy	7.99e^−05^	0.109	C8 is a constituent of the membrane attack complex. The gamma subunit seems to be able to bind retinol
*Pristimantis* Direct developers Elevation	100496214	MBL2	Single-copy	0.000112	0.232	Calcium-dependent lectin involved in innate immune defense. Binds to late apoptotic cells, facilitating their uptake by macrophages. May bind DNA
*Pristimantis* Direct developers	497005	LAMP2	Duplicated	0.000212	0	Plays an important role in chaperone-mediated autophagy, a process that mediates lysosomal degradation of proteins in response to various stresses
*Pristimantis*	100124807	NDUFA2	Single-copy	0.000394	0	Accessory subunit of the mitochondrial membrane respiratory chain NADH dehydrogenase (Complex I)
*Pristimantis*	448156	NECTIN2	Single-copy	0.000432	0.431	Probable cell adhesion protein. Modulator of T-cell signaling. Can be either a costimulator of T-cell function, or a coinhibitor, depending on the receptor it binds to
*Pristimantis*	548500	FMO5.2	Duplicated	0.0004689	0.1756	Involved in NRF2-mediated oxidative stress response, the unfolded protein response and response to hypoxia and cellular stress, indicating a role for the enzyme in adaptation to oxidative and metabolic stress. Also plays a role in stimulating a wide range of metabolic pathways and processes
*Pristimantis* Direct developers	100216255	C3	Duplicated	0.0005877	0.7019	C3 plays a central role in the activation of the complement system. Its processing by C3 convertase is the central reaction in both classical and alternative complement pathways.
*Orthogroups under intensified positive selection*						
*Pristimantis*	100492598	SIDT2	Duplicated	0	20.8677	Mediates the translocation of RNA and DNA across the lysosomal membrane during RNA and DNA autophagy
*Pristimantis* Direct developers	496992	FETUB	Duplicated	0	2.2739	Cysteine-type endopeptidase inhibitor activity
*Pristimantis* Direct developers	100491991	HAVCR1	Duplicated	2.22e^−16^	2.6006	Integral component of membrane
*Pristimantis*	549687	GOT2	Duplicated	8.88e^−16^	4.5247	Is important for metabolite exchange between mitochondria and cytosol, and for amino acid metabolism
*Pristimantis* Direct developers	448028	ODC1	Duplicated	6.46e^−12^	20.795	Catalyzes polyamine biosynthesis. Polyamines are essential for cell proliferation and are implicated in cellular processes, ranging from DNA replication to apoptosis.
*Pristimantis* Direct developers	780143	ACAA1	Duplicated	2.78e^−11^	5.001	Responsible for the thiolytic cleavage of straight chain 3-oxoacyl-CoAs lipid metabolism
*Pristimantis* Elevation	549716	NMRAL1	Duplicated	7.01e^−11^	10.408	Redox sensor protein
*Pristimantis*	448618	EIF4B	Duplicated	1.57e^−08^	1.059	Required for the binding of mRNA to ribosomes. Promotes the ATPase activity and ATP-dependent RNA unwinding activity of both EIF4-A and EIF4-F
*Pristimantis* Direct developers	448083	SULT1C1	Single-copy	6.03e^−08^	21.027	Sulfonate donor to catalyze the sulfate conjugation of drugs, xenobiotic compounds, hormones, and neurotransmitters
Elevation	394861	GNAI2	Single-copy	0.00000182	44.774	Involved as modulators or transducers in various transmembrane signaling systems
*Pristimantis* Direct developers	448709	APOA1	Single-copy	0.000198	23.092	Participates in the reverse transport of cholesterol from tissues to the liver for excretion as part of the SPAP complex, activates spermatozoa motility

For orthogroups significant in multiple hypotheses, the lowest RELAX *P*-value from the two analyses was selected. Relevant pathways or functional information for intensified and relaxed orthogroups were summarized from gene descriptions in iHypoxia database.

### Genes Associated With High Elevation

The majority of orthogroups under positive selection in *Pristimantis* were not under positive selection in the other two high-elevation species, *N. parkeri* (2,790 to 5,000 masl) and *S. sikkimensis* (2,700 to 4,200 masl). The relatively small overlap between significant orthogroups in the *Pristimantis* and high-elevation hypotheses suggests that there is a degree of genomic flexibility in amphibian adaptation to high altitudes, and that some mechanisms for responding to hypoxia may be species-specific (Lau et al. 2019). This has been observed in genome scans of Tibetan and Andean humans showing few common targets of selection despite their similar evolution at high elevations (Bigham et al. 2010). Uplift of the Andean mountain range is relatively recent compared to the Tibetan plateau where *N. parkeri* and *S. sikkimensis* inhabit, and so the amount of evolutionary time of since colonization of high-elevation habitats may account for our observed discrepancy in positive selection between *Pristimantis* and the other high-elevation species. The three orthogroups under selection only in the other high-elevation lineages (PCBD2, ANXA5, GNAI2), function in similar biochemical pathways as the additional 21 positively selected genes unique to *Pristimantis* (e.g. energy metabolism, cellular stress signaling, and coagulation), and have been identified in other studies of high-elevation species. For example, ANXA5, a well-known anticoagulant protein, has been repeatedly associated with altitudinal adaptations in antelope and deer mice (Ge et al. 2013; Schweizer et al. 2021), and was also recovered as a candidate gene under convergent positive selection in another study of high-elevation Tibetan amphibians including *N. parkeri* (Lu et al. 2020 ). Ectothermic species may have evolved different genetic mechanisms to cope with high-elevation environments compared to those in endotherms, so comprehensive functional studies of genes like ANXA5 will be required to better understand their general effects on performance of high-elevation amphibians (Yang et al. 2012).

We identified ten single-copy orthogroups under positive selection in *Pristimantis* which also bear signals of positive selection in one of the other high-elevation species, *N. parkeri* or *S. sikkimensis*. Their gene functions are related to redox processes (putative oxidoreductase), inflammatory response and cell signaling (MBL2, C8G, F11R), growth (FXR2, NNMT), and lipid metabolism (APOM, HSD17B13, ABHD15) (supplementary tables S4 and S7, Supplementary Material online). Several of these orthogroups were identified as candidate genes in other studies of hypoxia or highland adaptations in ectotherms (Wang et al. 2015; Yang et al. 2015), and their putative functions comprise important pathways known for responding to stresses from hypoxia or strong UV radiation. For example, lipid metabolism genes have been identified under positive selection in the Tibetan frogs and lizards (Sun et al. 2018), as well as burrowing caecilians (Torres-Sánchez et al. 2019). Lipid oxidation yields more energy than carbohydrates but requires more oxygen per mole of ATP synthesized, so positive selection in lipid metabolism pathways in ectotherms may accommodate efficient utilization of fats for ATP production to compensate for decreased metabolic output under hypoxia, as observed in the Tibetan lizard *Phrynocephalus erythrurus* (Tang et al. 2013).

Overall, our identified candidate genes under positive selection across high-elevation frogs are consistent with previous studies in suggesting that changes to their associated functions represent important convergent evolutionary adaptations to high-altitude life. However, given that we have a relatively small sample size of three high-elevation amphibian clades and the non-exclusivity of the high-elevation hypothesis, lineages, additional genes enabling high-elevation adaptation may be evolving under selection that were not captured by our species in the tested foreground. Furthermore, it is likely that patterns of selection found in high-elevation Andean *Pristimantis* lineages in Ecuador do not recapitulate adaptive evolution patterns in *Pristimantis* as a whole, as many *Pristimantis* do not live at high elevations. Adaptation to high elevations has possibly evolved multiple times independently within *Pristimantis*. Our current study does not have the sampling breadth to differentiate if the observed patterns of selection are genus-wide or instead pertain specifically to high-elevation *Pristimantis* species and lineages. More comprehensive analysis of molecular indicators of selection in *Pristimantis* and other high-elevation clades will require further sequencing studies on amphibians.

### Genetic Basis of Direct Development

When we included *O. cruralis* (the single non-*Pristimantis* direct-developing species in our dataset) in the foreground for our selection tests, we identified some genes under positive selection that were also under positive selection but not significant within the *Pristimantis* clade only. One explanation for this pattern is that these sequences have differentiated in direct developers compared to other frog species to yield functional innovations to accommodate lacking a tadpole life stage. For instance, we observed unique positive selection in dioxygenases and genes that regulate production of reactive oxygen species (ROS) (supplementary table S4, Supplementary Material online; Fig. 2). These genes could have specialized roles for direct development by assisting in limb formation or growth with increased levels of molecular oxygen and ROS production in terrestrial environments compared to less-oxygenated water. In the best-studied direct developer, *Eleutherodactylus coqui*, limb formation within terrestrial eggs is characterized by accumulation of ROS that increase interdigital cell death (ICD), and direct development has been proposed to be evolutionarily and functionally linked to increased oxygen availability within the limbs (Cordeiro and Tanaka 2020). Given that we only have one additional direct developer in our dataset (*O. cruralis*) that is sister to the *Pristimantis* clade, another possible explanation for our findings is that these genes under selection reflect early changes facilitating *Pristimantis* or other Terrarana frogs’ success, rather than relating specifically to terrestrial development within eggs. For some orthogroups that narrowly missed the threshold for significance in the *Pristimantis* hypothesis, including the additional neighboring foreground branch (the other direct developer *O. cruralis*) moved likelihood scores below the significance threshold. In such a case, selection is happening in both hypotheses, but the significant gene is identified only when the larger sample size, including the *O. cruralis* branch, is analyzed. For example, some direct developer genes under selection are involved in iron and other metal ion binding (ETHE1, SHDC, BCO2), which is functionally consistent with different genes under selection within *Pristimantis* that modulate redox status and mitochondrial functions or attenuate pulmonary vascular response to acute hypoxia (Smith et al. 2008).

We also identified 41 candidate genes for direct development under positive selection both within the *Pristimantis* clade and across all direct developers. For example, fibronectin (FN1) sequences form a monophyletic gene tree clade in direct developers compared to other frog species, and we obtained much stronger support values for FN1 positive selection when *O. cruralis* was included in the foreground compared to testing *Pristimantis* alone (BUSTED adj. *P* = 2.39e^−12^ vs. 0.0002, respectively; supplementary tables S8 to S10, Supplementary Material online). Fibronectins are adhesion proteins that have been found to play a role in embryogenesis and cell differentiation within developing *Xenopus* embryos, where they connect cells via extracellular matrices that form tissues (Smith et al. 2009). Another candidate gene is FETUB (fetuin B), which had stronger support for intensified positive selection (RELAX *P* = 0) in direct developers compared to just *Pristimantis* (RELAX *P* = 0.00281). The mammalian homologs of fetuin B are liver-derived plasma proteins of the cystatin-superfamily that inhibit ovastacin to harden the zona pellucida, making them essential for female fertility (Karmilin et al. 2019). Astacin metallopeptidase paralogs are also present in lower vertebrates, so through intensified positive selection, fetuin B could have evolved an analogous role in reproduction in *Pristimantis*. Neither FN1 nor FETUB have been reported to evolve under positive selection in high-elevation species, enhancing the plausibility of their role in direct development.

Our findings suggest that positive selection in genes like FN1 and FETUB may have led to alternative functions in anatomical structure organization and differentiation in direct developers. However, the non-exclusivity of the *Pristimantis* and direct developer hypotheses makes it difficult to pinpoint candidate genes important for direct development rather than adaptive processes in *Pristimantis*. Ultimately, because our dataset contains only one other direct developer that is sister to the *Pristimantis* clade, we were unable to compare selection in independent lineages where the tadpole stage has been lost. The shared ancestry between *Pristimantis* and *O. cruralis* could be sufficient enough to explain the overlapping genes identified in the *Pristimantis* and direct developer hypotheses. Thus, without additional foreground branches to strengthen our analysis, we cannot definitively conclude that genes under selection are crucial for evolutionary adaptation to terrestrial egg-laying instead of other factors not considered in this study. Additionally, the lack of sequences from other direct-developing frogs may have caused us to miss important candidate genes. For example, ATRAID, which is involved in cellular differentiation and bone mineralization, was under positive selection in *Pristimantis*, but an orthologous sequence was not recovered for *O. cruralis* so the hypotheses test results could not be compared. Thus, generating additional sequences from other direct-developing frog species will be essential for future studies verifying functional roles of these genes and whether they are involved in direct development.

### Gene Functions Under Selection in *Pristimantis*

Separate from the genes and pathways discussed above that were positively selected in both *Pristimantis* and the other high-elevation species, we identified six single-copy genes known to affect vascular tissue remodeling, growth, and cell differentiation that were significant only within the *Pristimantis* clade (supplementary table S4, Supplementary Material online). For example, ADTRP disrupts the tissue factor-dependent pathway of coagulation by disrupting thrombin production (Lupu et al. 2021), and CREG1 is a secreted protein involved in cardiomyogenesis that affects growth of cells during vascular remodeling (Li et al. 2017). Additionally, a hemoglobin subunit (HBA2) was uniquely under positive selection in *Pristimantis*. Evolutionary changes in hemoglobin structure and oxygen affinity are well-studied parts of adaptation to high altitudes because efficient oxygen loading and transport in the blood is essential for survival under hypoxia (Storz et al. 2013). High-elevation Andean frog species (Weber et al. 2002), black-spotted frogs (Ma et al. 2023), and Asiatic toads (Pu et al. 2021) have higher blood oxygen affinities and smaller erythrocyte volumes compared to lowland relatives. Amino acid substitutions in the HBA2 region may indicate similar properties in *Pristimantis* or changes in blood composition to reduce viscosity and hypertension in hypoxic environments. The HBA2 orthogroup was not tested in all high-elevation species due to missing a *S. sikkimensis* sequence, but previous studies surprisingly did not find hemoglobin genes under selection in Tibetan frog species, including *N. parkeri* (Lu et al. 2020; Yang et al. 2012). In light of this, we suggest that hemostatic properties warrant further investigation for an adaptive role specific to *Pristimantis* lineages in the Andes.

The functional annotation cluster from DAVID with the largest number of genes under positive selection in *Pristimantis* is comprised of genes involved in mitochondrial processes related to energy metabolism (Fig. 2), which is unsurprising given that mitochondrial genes have been repeatedly linked to adaptation in other high elevation taxa (Lim et al. 2019; Szpiech et al. 2021). At higher elevations with less oxygen, energy production from oxidative metabolism is reduced, so amino acid substitutions in positively selected *Pristimantis* genes like cytochrome C oxidase, ATP synthase, and NADH dehydrogenase subunits may function in modulating mitochondrial complexes for more efficient electron transport and heat production. Alternatively, these genes could have evolved to suppress metabolic demand and therefore required oxygen (Bickler and Buck 2007). Another potential issue at high elevations is accumulation of ROS and oxidative intermediates in mitochondria causing oxidative stress (Ge et al. 2015). We found positive selection in known antioxidant genes (SELENOS, PRDX5, SOD1) which may indicate their evolution for more efficient antioxidant activity or free radical scavenging. For example, a recent study found genes involved in inflammatory response, autophagy, and antioxidant metabolism displayed significant up-regulation in *N. parkeri* when exposed to UV radiation, preserving skin integrity and function compared to other frog species (Fu et al. 2022). Differences in metabolic output of aerobic respiration between amphibian species have not been investigated so far, and functional consequences of changes to energy production genes should be the focus of future studies.

Also prominently under selection in *Pristimantis* were genes involved in innate immune response pathways (multiple members of the complement system) and transmembrane signaling receptors affecting Ca^2+^ influx, autophagy, and endoplasmic reticulum stress (Fig. 2; supplementary table S4, Supplementary Material online). Immune system functions in ectotherms are strongly affected by temperature and other environmental stress factors (Raffel et al. 2006), so some of these genes may be under selection in *Pristimantis* to minimize immunosuppression from lower temperatures, hypoxia, and UV radiation at high elevations. For example, complement proteins play a crucial role in the innate immune response against infections and environmental stressors by recognizing and eliminating pathogens, enhancing phagocytosis, and triggering inflammation. We found several complement system proteins under positive selection (MBL2, C3, C8G, CB) which elicit pro-apoptotic and inflammatory effects in response to hypoxia (Mueller-Buehl et al. 2021), and have higher presence in plasma proteomes of humans resistant to acute mountain sickness (Sharma et al. 2022). They are also known to promote tissue homeostasis, angiogenesis, and pulmonary vascular remodeling (Bauer et al. 2011; Ricklin et al. 2010), which may confer adaptive benefits in high-elevation *Pristimantis* species. Furthermore, one of the genes under positive selection in all three hypotheses, mannose-binding lectin (MBL2), encodes a protein that synergizes with the complement system by recognizing damage-associated molecular patterns (DAMPs) to mediate release of proinflammatory cytokines in endothelial tissue and further complement activation (Garred et al. 2016). Another possibility is that *Pristimantis* immune genes may have diversified due to disparate selective pressures from environmental pathogen or microbial threats as *Pristimantis* colonized new habitats in the Andes (Warne et al. 2016). The contribution of immunity genes to *Pristimantis* evolution is still unknown and will require functional validation to be explored.

### Relaxed Purifying Selection in *Pristimantis*

The vast majority of single-copy genes under selection in *Pristimantis* are evolving under a regime of relaxed purifying selection (Fig. 3). Similar trends have been identified in other rapidly diversifying taxa (Schneider et al. 2019), but the precise significance of our results remains unclear. “Genes that are only expressed in specific environments are more susceptible to relaxed selection when organisms transition to non-inducing environments” (Pfennig et al. 2010). At high elevations, where environmental conditions differ significantly from those at lower altitudes, genes under strong purifying selection in lower altitudes may experience reduced selective pressure because their environmentally driven expression does not confer an advantage. Consequently, over time relaxed selection may lead to decreased importance of these genes for individual fitness and survival at high elevations. Furthermore, the release of selective constraints on these genes could contribute to the remarkable morphological diversity observed in *Pristimantis* species distributed across elevational gradients (Páez and Ron 2019). When organisms encounter novel non-inducing environments with available niches and resources, the adaptive landscape tends to flatten, and selective pressure on previously fixed ecological traits is relaxed. This relaxation of selective pressure can be expected to increase phenotypic variety as more phenotypes become viable. Another possibility is that relaxed purifying selection may indicate genomic evolution of phenotypic plasticity in *Pristimantis*, which is a strategy for coping with hypoxia in other taxa like the Tibetan zokor (Cai et al. 2018) and cichlids (Crocker et al. 2013). Specifically, relaxed selective constraint on protein-coding genes can facilitate them to more readily adopt innovative expression patterns that give rise to specialized phenotypes with improved fitness under specific conditions (Hunt et al. 2011). Plasticity could provide adaptive benefits in *Pristimantis*, for example, by altering their metabolism according to changes in environmental oxygen levels across elevations. Plastic responses to hypoxia can also be maladaptive, in which case plasticity would be selected against in favor of phenotypes with attenuated environmental sensitivity (Storz and Scott 2021). Examples of this include the evolution of blunted hypoxic pulmonary vasoconstrictive responses to hypoxia in some high-altitude human populations (Brutsaert et al. 2005). Fully inferring the functional significance of relaxed purifying selection in *Pristimantis* genes and whether gene expression can induce plastic phenotypic changes is beyond the scope of this study. Future work could be invaluable in this respect, perhaps by testing for differential expression of genes under relaxed selection among *Pristimantis* at different altitudes.

### Fate of Duplicated Genes in the Context of Adaptation

Orthogroups that were duplicated within *Pristimantis* were more likely to be under positive selection compared to single-copy genes, but we found no evidence that duplicated orthogroups were more likely to experience relaxed purifying selection or intensified positive selection for any of our tested hypothesis. Instead, we observed that single-copy genes were predominantly governed by relaxed purifying selection, experiencing less pressure to maintain their current form. In contrast, duplicated genes experienced increased selective pressure to change amino acid composition and thus function (i.e. intensified positive selection) (see Relaxed Purifying Selection in *Pristimantis*, Fig. 3). These findings may suggest that functional divergence after small-scale gene duplication is an adaptive mechanism in *Pristimantis*. Increased positive selection and neofunctionalization has been demonstrated to be the fate of young duplicates in diverging ancestors of mammalian lineages, changing from redundant copies to distinct genes with essential functions (Assis and Bachtrog 2015). Additionally, stronger positive selection after duplication of certain genes may be generally beneficial for high-altitude adaptation in amphibians and not limited only to *Pristimantis*. Out of seven orthogroups that were duplicated in *Pristimantis*, *S. sikkimensis*, and *N. parkeri*, only the NMRA-like redox sensor (NMRAL1) was significant for intensified positive and diversifying selection in all three high-elevation lineages (RELAX and aBSREL, supplementary table S10, Supplementary Material online). NMRAL1 plays a role in the synthesis of nitric oxide and regulating redox homeostasis and was recently identified as a candidate adaptation gene in high-elevation breeds of goats (Manunza et al. 2023). Duplication and intensified positive selection of NMRAL1 in all high-elevation frog lineages are thus intriguing and invites further study of the contribution of natural selection to adaptive functional evolution and long-term fate of duplicate genes.

Duplicated orthogroups were significantly enriched for macroglobulin-like coding domains associated with innate immune response (supplementary fig. S5, Supplementary Material online). Expansion of immunity-related domains has been theorized to facilitate adaptation in diverse animal lineages by improving stress responses and pathogen recognition as they expand into new habitats (Flajnik 2018), so similar mechanisms may be at play in *Pristimantis*. We also identified a potential role for complement gene family expansion in that more copies of complement C5 protein were significantly correlated with species elevation in *Pristimantis* (Fig. 5). Complement C5 contributes to activation of neutrophils and leukocytes involved in inflammatory response and C5a, an anaphylatoxin produced when complement is activated, is a chemoattractant that recruits immune cells to the site of infection or injury. In conjunction with our findings that several complement genes are under significant positive selection (Tables 1 and 2, supplementary material S1, Supplementary Material online), the correlation highlights the potential importance of complement proteins in responding to elevation stress. Although we did not find a significant relationship between species elevation and higher dN/dS values for any of the orthogroups tested, having more copies of genes like C5 may allow *Pristimantis* species to better control stress responses across high-elevation environments by dynamically influencing mRNA and protein expression levels (Kondrashov 2012; Schlattl et al. 2011). However, this relationship may be specific to this particular species group, and expanding the scope of this research with larger sample sizes and broader taxonomic coverage is crucial to confirm and generalize these findings in other *Pristimantis* species. Another distinct possibility is that these sequences may not be true duplicates, but rather isoforms generated from alternative splicing. Alternative splicing events are prevalent in nervous and immune system genes (Chang and Zhang 2017) and mainly serve to increase the diversity of mRNA expressed from the genome. Changes from alternative splicing are usually small but can generate functional effects when induced in a coordinated fashion, for example, by differentially regulating gene expression in hypoxia response for high-altitude pigs (Yang et al. 2022). The true nature of these sequences can be addressed in future studies by assessing divergence in gene expression (Sandve et al. 2018) or validating alternative splicing events among these duplicate sequences in multiple *Pristimantis* species.

Our phylogenetic comparison of the first *Pristimantis* transcriptomes to orthologs expressed in other frog species extends our understanding of their evolutionary diversification. We found that candidate adaptation genes under selection in *Pristimantis* are related to mitochondrial oxidative respiration, hemostasis, cellular signaling processes, and innate immune response. Positive and relaxed purifying selection in these genes likely resulted in sequence substitutions that confer adaptive benefits to *Pristimantis* frogs during species diversification and niche colonization in the Andes. Orthogroups duplicated within *Pristimantis* are more likely to be evolving under positive selection but not relaxed purifying selection, suggesting that some duplicated gene copies evolving under intensified positive selection may have ecologically relevant functions across *Pristimantis* species. While specific functional consequences of *Pristimantis* genes under selection are beyond the scope of this study, the genes and pathways identified under selection add context to what we know about amphibian and high-elevation molecular evolution, providing candidate sets of expressed genes for further examination in frogs and other highland taxa.

## Methods

### Taxon Sampling

A total of 41 anuran species were included in our analysis (supplementary table S3, Supplementary Material online, Fig. 1a). We downloaded transcriptome assemblies from 34 anuran species published in the NCBI Transcriptome Shotgun Assembly and NCBI Refseq databases. Our goal was to use the largest assembled transcriptomes with the most complete sequences across anurans and covering the greatest diversity of genera. When possible, we chose transcriptomes taken from several tissues to obtain the most complete sets of transcripts. This dataset included species inhabiting elevations ranging from 0 to 5,000 m above sea level, and only one species, *O. cruralis*, is a direct developer. For our final dataset, we added de novo transcriptomes from seven *Pristimantis* species, all of which are direct developers and occupy high-elevation ecosystems.

*Pristimantis* species were collected under the permit MAE-DNB-CM-2015-0017, issued by the Ministerio de Ambiente, Ecuador. We collected and sampled liver tissue from seven individuals in the genus *Pristimantis*. These individuals belong to two formally described species (*Pristimantis orestes and Pristimantis andinognomus*) and five putative species that currently lack formal denomination, but are distinct species based on phylogenetic analysis of three gene fragments (Urgiles et al., in prep), and will be hereafter characterized as *P. sp1*, *P. sp2*, *P. sp3*, *P. sp4*, and *P. sp5*. Samples were collected across six different localities that range between 2,500 and 3,500 m of elevation in the southern Ecuadorian Andes. Specimens were collected at night during the last 2 weeks of May 2019 in hours between 7 PM and 10 PM. Collected individuals were placed in plastic bags with dampened paper towels and transported to the Zoology Museum at Universidad del Azuay, Cuenca, Ecuador. Specimens were euthanized with 2% lidocaine, and immediately afterwards a piece of liver was obtained through an abdominal incision and placed in a vial containing RNAlater. All samples were maintained in a −80 °C freezer.

### RNAseq and Transcriptome Assembly

We isolated total RNA from specimens using TRIzol (Invitrogen, MA) following the manufacturers protocol and used the Agilent TapeStation with RNA ScreenTapes to evaluate sample quality (RIN scores) and concentration. We used the Dynabeads mRNA Direct kit (Invitrogen/Life Technologies 610.11/12) to isolate mRNA molecules by 3′ poly-A capture. We converted extracted RNA into double-stranded complementary DNA (cDNA) using the Superscript III First Strand Synthesis System (Invitrogen/ABI #18080-051) with random hexamers and the NEBNext mRNA Second strand Synthesis Module (New England Biolabs # E611S/L). We used magnetic speed beads (Cytiva 65152105050250a) to clean and remove small fragments and residual reagents from samples after all subsequent steps. We sheared the cDNA into 300 bp fragments with a Covaris sonicator. We blunted the fragmented cDNAs with a 1 mM dNTP mix and DNA Polymerase I, Large (Klenow) Fragment (NEB, # M0210S), then generated 3′ cytosine overhangs using 10× NEB Buffer 2 with dCTP and Klenow Fragment (3′–5′ exo-) (NEB # M0212S). Stubby y-yoke adapters with G overhangs (Glenn et al. 2019) were then ligated to the C-tailed fragments using 1 μl of T4 DNA ligase (NEB, M0202S), 10 μl of 5× Quick Ligation Buffer, 1 μl of 5 nM Stubby, and 13 μl of double-distilled water per library. We dual-indexed each library using unique combinations of i5 and i7 Illumina adapters with 8 bp indexes (Glenn et al. 2019). Adapters were added via limited cycle PCR using 25 μl of Kapa HiFi Master Mix (Kappa Roche), 2.5 μl of each 10 μM i5 and i7 barcoded adapters, 10 μl of double-distilled water, and 10 μl of DNA and Kapa HiFi Taq (Roche) per reaction. The PCR conditions were as follows: initial step for 45 s at 98 °C, followed by 20 cycles of denaturation for 10 s at 98 °C, annealing for 30 s at 60 °C, and extension for 30 s at 72 °C; with a final extension for 3 m at 72 °C. Each library was quantified with KAPA Library Quantification Kits (Roache), equimolarly pooled, then the final pool was re-quantified. Libraries were sequenced on the Illumina HiSeq platform using 2 × 150 bp chemistry. We assessed raw reads using FastQC (Andrews 2010) and performed quality trimming and adapter removal using Trimmomatic (Bolger et al. 2014). We assembled one de novo transcriptome for each *Pristimantis* species using Trinity with default parameters (Haas et al. 2013).

### Orthogroup Construction

We used TransDecoder (http://transdecoder.github.io) to accurately predict open reading frames (ORFs) from the transcriptomes of the 41 tested frog species. We removed redundant transcripts from transcriptomes using CD-HIT with a sequence similarity threshold of 0.9 (Fu et al. 2012). While this approach does not guarantee certainty in distinguishing true duplicates from isoforms, it significantly enhances the likelihood of removing spurious isoforms and identifying genuine duplicate sequences in our analysis. We ran Orthofinder (Emms and Kelly 2015) with default parameters on the output translated coding domain files to cluster protein sequences into orthologous groups (hereafter, orthogroups). For selection analyses and species tree construction, we used PhyloPyPruner (Kocot et al. 2013) to prune paralogous sequences from our orthogroup alignments and remove alignments with fewer than 20 of our species or which retained no sequences from *Pristimantis* after pruning. Pruned orthogroup protein sequences retained by PhyloPyPruner were re-aligned using MAFFT (Katoh and Standley 2013), and gene trees were reconstructed for every orthogroup using IQ-TREE (Nguyen et al. 2015). We used the subseq function from seqtk (https://github.com/lh3/seqtk) to generate nucleotide fasta files for each protein orthogroup file and produced codon-aware alignments using the protein alignments as guides with PAL2NAL (Suyama et al. 2006). We removed any trailing stop codons from nucleotide alignments by running removestopcodons.bf within Hyphy (Kosakovsky Pond et al. 2020).

### Anuran Phylogeny Reconstruction

To generate our final species tree, we ran IQ-TREE inputting the concatenated amino acid sequence alignments retained by PhyloPyPruner while setting the *Bombina* species as the outgroups (Nguyen et al. 2015). For our parameters, we used amino acid models with 5,000 ultrafast bootstrap replicates. We then re-ran Orthofinder setting the rooted tree generated by IQ-TREE as the final defined species tree (“-s, -fg” options), so Orthofinder would more accurately infer duplication events for individual orthogroups within *Pristimantis* and direct developers. We used this species tree for all phylogenetic regressions and for plotting values of selection across species. We generated a midpoint-rooted version of this phylogeny for ease of visualizing species relationships (Fig. 1).

### Functional Annotation of Orthogroup Sequences

We used the blastp function in DIAMOND to compare orthogroup sequence files from Orthofinder against the Uniref90 database (Buchfink et al. 2021). We used InterproScanV5 (Jones et al. 2014) to look for common Pfam domains and assign GO terms and imported them with Kinfin for orthogroup-level annotations (Laetsch and Blaxter 2017). Orthogroups without matches from either InterproScan or DIAMOND were excluded from further analyses due to lack of information about the sequences. We additionally ran BUSCO on each species’ transcriptome to quantify the proportions of conserved and complete orthologous groups from the metazoan BUSCO database (Simão et al. 2015). We used PANNZER to find official gene symbols and more specific GO terms for each orthogroup inferred to be under positive selection (Törönen and Holm 2022), and used OrthoDB (Kuznetsov et al. 2023) to obtain Entrez IDs of associated *Xenopus tropicalis* orthologs. For each hypothesis, we submitted the IDs of positively selected genes to the DAVID web database (Sherman et al. 2022) and used the Functional Annotation Clustering function to identify overrepresented biological terms under selection (FDR < 0.05) and group functionally related KEGG, REACTOME, and GO annotation terms to focus on biological interpretation of these terms at the group level. Lastly, we searched all genes under selection in the iHypoxia database (Liu et al. 2023) to check if they have been previously connected to hypoxia response or adaptation to high elevations in other species, and thus are potentially adaptive in *Pristimantis*.

### Orthogroup Selection Analyses

For all selection analyses, we ran each program separately with the foreground on the gene tree selected as (i) just the *Pristimantis* clade, (ii) the direct developer clade (*Pristimantis* clade + *O. cruralis*), or (iii) all high-elevation species (*Pristimantis* clade + *S. sikkimensis* + *N. parkeri*). We used two programs from the Hyphy package (Kosakovsky Pond et al. 2020) to analyze our orthogroup sequence alignments and gene trees for significant evidence of positive selection. We first used BUSTED (Branch-Site Unrestricted Statistical Test for Episodic Diversification) to test whether a given gene has been subject to positive selection at any point in the specified branches (Murrell et al. 2015). We corrected the BUSTED *P*-values to obtain FDR among multiple comparisons using Benjamini–Hochberg (2010). We used a paired *t*-test to infer if average *ω* (dN/dS) values generated by BUSTED were different between foreground and background branches. We also used the branch-site model aBSREL (adaptive Branch-Site Random Effects Likelihood), which infers the number of *ω*-rate categories along each gene tree branch and used the output to evaluate the prevalence of episodic diversifying selection in specific lineages and individual branches (Smith et al. 2015). We conducted principal components analysis (PCA) using the prcomp function in R to summarize the variance of omega values and modeled the distribution of the first principal component values (PC1) on our species tree using the “plotBranchbyTrait” function from phytools (supplementary fig. S4A to C, Supplementary Material online). In addition to Hyphy analyses, we ran CODEML from the PAML package (Yang 2007) on the orthogroup alignments using the branch-site model (Model A/A1). We ran CODEML on each alignment twice, to compare likelihood scores for the detection of positive selection along two or more branches under the alternate model (A1), where *ω* (omega = dN/dS) is allowed to vary, and the null model (A), where *ω* is constrained to neutral evolution (*ω* = 1) (Bielawski et al. 2016). We used a chi-squared test to compare the log-likelihood scores from both fitted models for significant differences. We corrected the CODEML *P*-values to obtain FDR among multiple comparisons using Benjamini–Hochberg (2010).

To test for relaxed or intensified selection for our three tested foreground hypotheses, we ran RELAX (Wertheim et al. 2015) on all orthogroups that were found to be under positive selection from all three selection tests (BUSTED, aBSREL, CODEML). Under the RELAX model, the strength of purifying (*ω* < 1) and positive (*ω* > 1) selection can both be intensified if *ω* values across test branches are driven further away from the neutral expectation of 1 compared to background, with decreasing and increasing *ω* representing intensified purifying and positive selection, respectively. Likewise, purifying selection is relaxed if lower *ω* values (0 < *ω* < 1) increase toward 1 in test compared to background branches, and positive selection is relaxed when higher *ω* values (*ω* > 1) decrease toward 1 (see supplementary fig. S1, Supplementary Material online). We used a two-sided Fisher's Exact Test to test for differences in the number of orthogroups experiencing relaxed versus intensified selection in *Pristimantis*, direct developers, and high-elevation species relative to all other species. We conducted PCA to summarize the variance of the *k* relaxation parameter values (exponent of dN/dS, *k* in *ω^k^*) across species for all orthogroups identified by RELAX as having significant evidence of relaxed or intensified selection (*P* ≤ 0.05). To visualize which clades experienced changes in selection strength, we plotted PC1 scores estimated from these significant *k*-values on our species tree (supplementary fig. S4A to C, Supplementary Material online). We used a two-sided Wilcoxon signed-rank test to infer whether the overall *k*-value distribution differed significantly from the null expectation of 1 (*k* = 1, neutral selection) for our three tested foreground hypotheses. We submitted annotations of significantly intensified or relaxed orthogroups to the Reactome pathway knowledgebase (Gillespie et al. 2022) to generally classify their molecular functions.

### Duplicated Orthogroup Selection Analyses

We tested for signals of selection in duplicated orthogroups whose expansion could be related to diversification in the context of our three tested foreground hypotheses. We analyzed these orthogroups separately from single-copy ones because PhyloPyPruner removes any gene trees and alignments thought to contain paralogous duplicates. To address this, we instead extracted the longest sequence for each species from duplicated Orthofinder protein alignments, so that each alignment contained one representative sequence. Using this approach allowed us to test selection among duplicated sequences without significantly increased computational demands from using entire orthogroup gene trees. We repeated all previously described selection analyses (CODEML, BUSTED, aBSREL, and RELAX, see Phylogenetic Tests of Relationships Between High-Elevation Dwelling and Orthogroup Duplications), this time using only orthogroups that were inferred to be duplicated by the STRIDE (Emms and Kelly 2017) algorithm in Orthofinder (support likelihood value ≥ 0.5) at species tree nodes within *Pristimantis*, within the direct developer clade, and duplicated within *Pristimantis* and the other high-elevation species (supplementary fig. S6, Supplementary Material online). Orthogroups without matches from either InterproScan or DIAMOND were excluded from further analyses due to lack of information about the sequences. We used an unpaired *t*-test to see if average *ω* values differed significantly between duplicated- and single-copy genes for each foreground hypothesis. We tested for overrepresentation of GO terms in orthogroups which STRIDE found to be duplicated within our tested tree clades using a hypergeometric test in R to identify and visualize significantly overrepresented GO terms and Pfam domains (FDR < 0.05) within duplicated orthogroups against a gene background of all annotated orthogroups.

### Phylogenetic Correlations Between Duplicated Gene Copies and Elevation

We tested for correlations between the number of sequences within duplicated orthogroups and species elevation using PGLS with our constructed phylogeny to account for the nonindependence of our frog species due to relatedness (Martins and Hansen 1997). We only used these orthogroups because we were interested in evaluating gene families that expanded within the *Pristimantis* clade as an explanation of increasing adaptation to high elevation, instead of identifying general relationships between gene counts and elevation. The mean elevation estimations for each frog species were obtained using lower and upper ranges available in AmphibiaWeb (https://amphibiaweb.org), Amphibians species of the world (Frost 2014), and the IUCN red list of threatened species (IUCN 2024) web sources. Because the taxonomy of *Bufotes viridis* is complex, we used elevational ranges exclusively from *B. viridis viridis* populations according to Borkin et al. (2000). We recorded the elevation at which we collected each of the *Pristimantis* frogs included in this study in the field with a GPS. For our predictive variable, we used transcript counts from the “Gene count” file generated from Orthofinder. We used the gls function from the nlme package in R and specified a generalized linear mixed model with elevation as our predictive variable among frog species and transcript counts as the response variable. To account for the size disparity of transcriptomes in our dataset, we included a term in the model representing the total number of sequences in each species’ transcriptome. We ran this analysis for each orthogroup and adjusted for multiple testing by applying sequential Bonferroni–Holm correction. To test if orthogroup duplications may be related to adaptation to higher elevation within *Pristimantis* specifically, we also ran these analyses using only the *Pristimantis* clade.

In addition to gene counts, we also tested whether positive selection in the duplicated orthogroups is correlated with species elevation. Because changes in selective pressure are more likely to follow Brownian motion, we used the gls function from the nmle R package with a Brownian model of evolution. We regressed PC1 scores from *ω* values from aBSREL and percentages of sites under selection as response variables with the elevation for each species as the predictive variable.

## Supplementary Material

evae167_Supplementary_Data

## Data Availability

The sequence reads generated in the current study will be deposited in NCBI's Short Read Archive upon manuscript acceptance. The scripts developed for analysis can be publicly accessed at https://github.com/Nchristo-Bioinformatics/frog_orthogroups_workflow/. The data underlying this article are available in the Dryad Digital Repository, at https://datadryad.org/stash/share/Owck5QRy9UzDzO9psgR1lQ–oLjSJekDLannWj6ttv40. Raw sequence reads are deposited in the NCBI SRA database (BioProject PRJNA1146027).
