# Supplementary material for: Selection and Gene Duplication Associated With High-Elevation Diversification in Pristimantis, the Largest Terrestrial Vertebrate Genus
Source: Genome Biol Evol. 2024 Aug 7;16(8):evae167. doi: 10.1093/gbe/evae167 (PMC11342244; doi:10.1093/gbe/evae167)
Supplement: evae167_Supplementary_Data [file evae167_supplementary_data.zip › Supplementary_figures_final_6_18.docx]

**Supplementary Figures for “****Selection and gene duplication associated with high-elevation diversification in *Pristimantis*, the largest terrestrial vertebrate genus”**


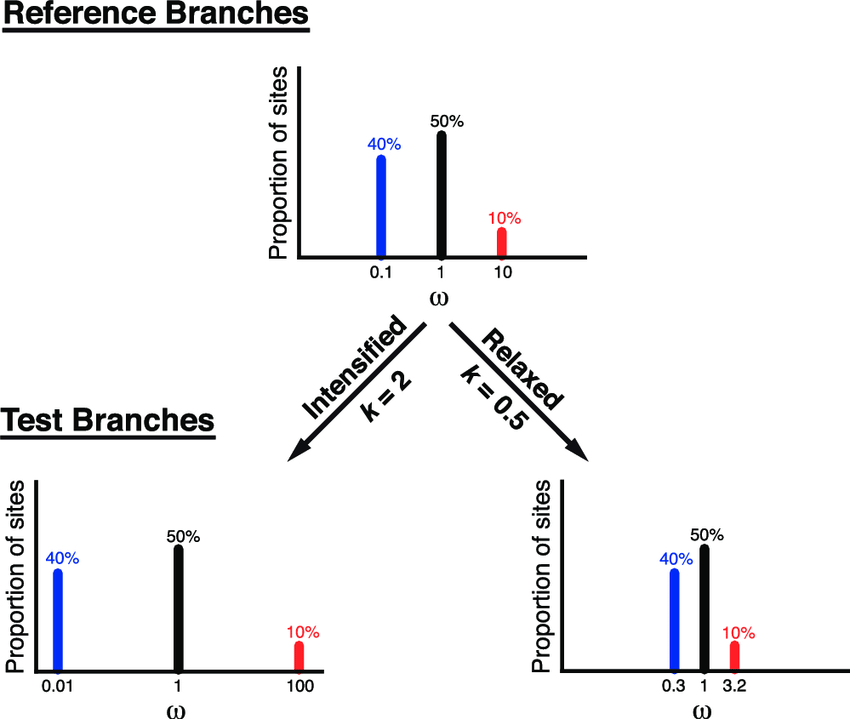


**Supplementary Figure 1.** RELAX dN/dS distributions under relaxed and intensified selection.


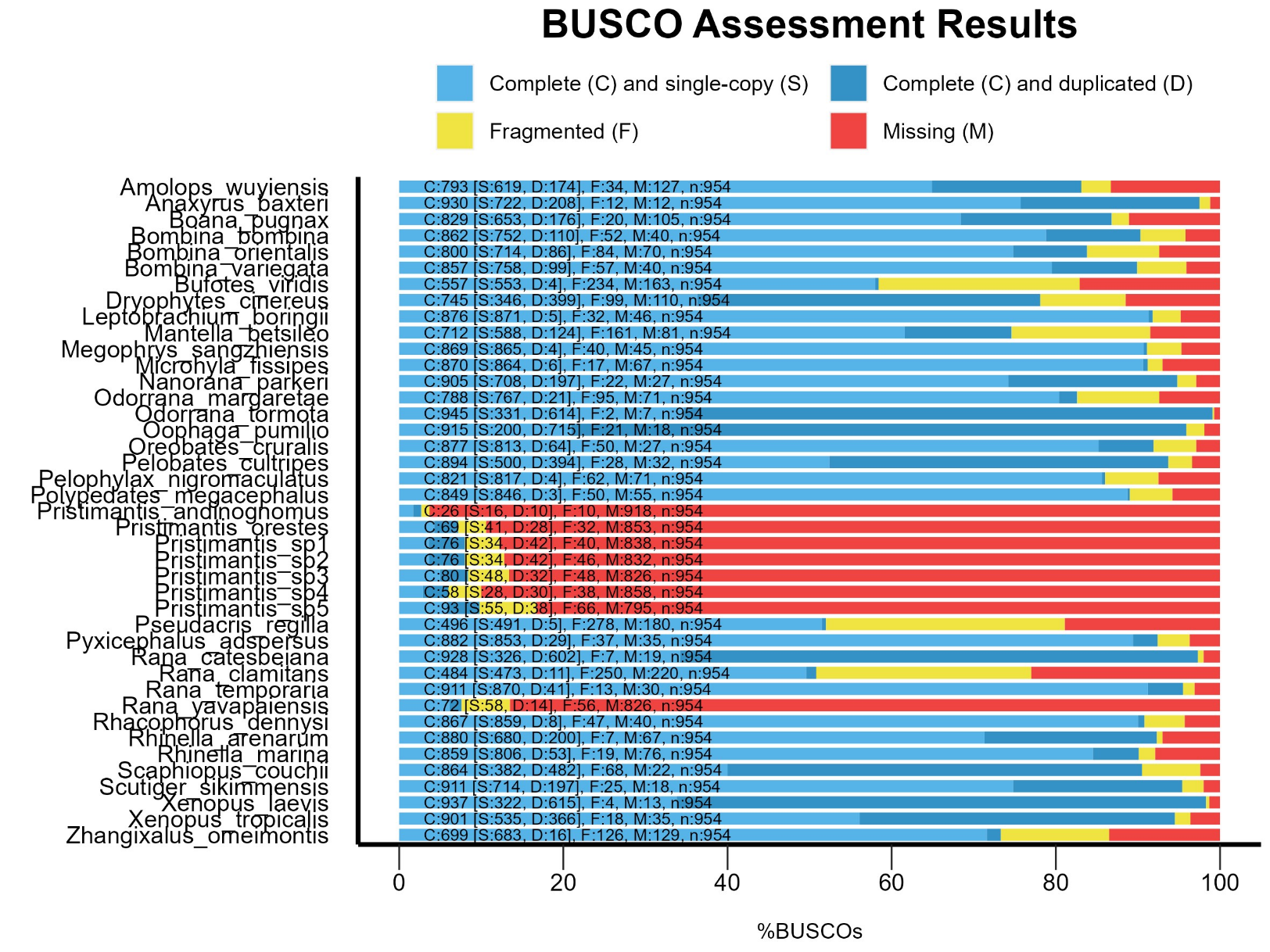


**Supplementary Figure 2.** Missing and fragmented BUSCO comparisons among *Prismantis* and other frog transcriptomes.


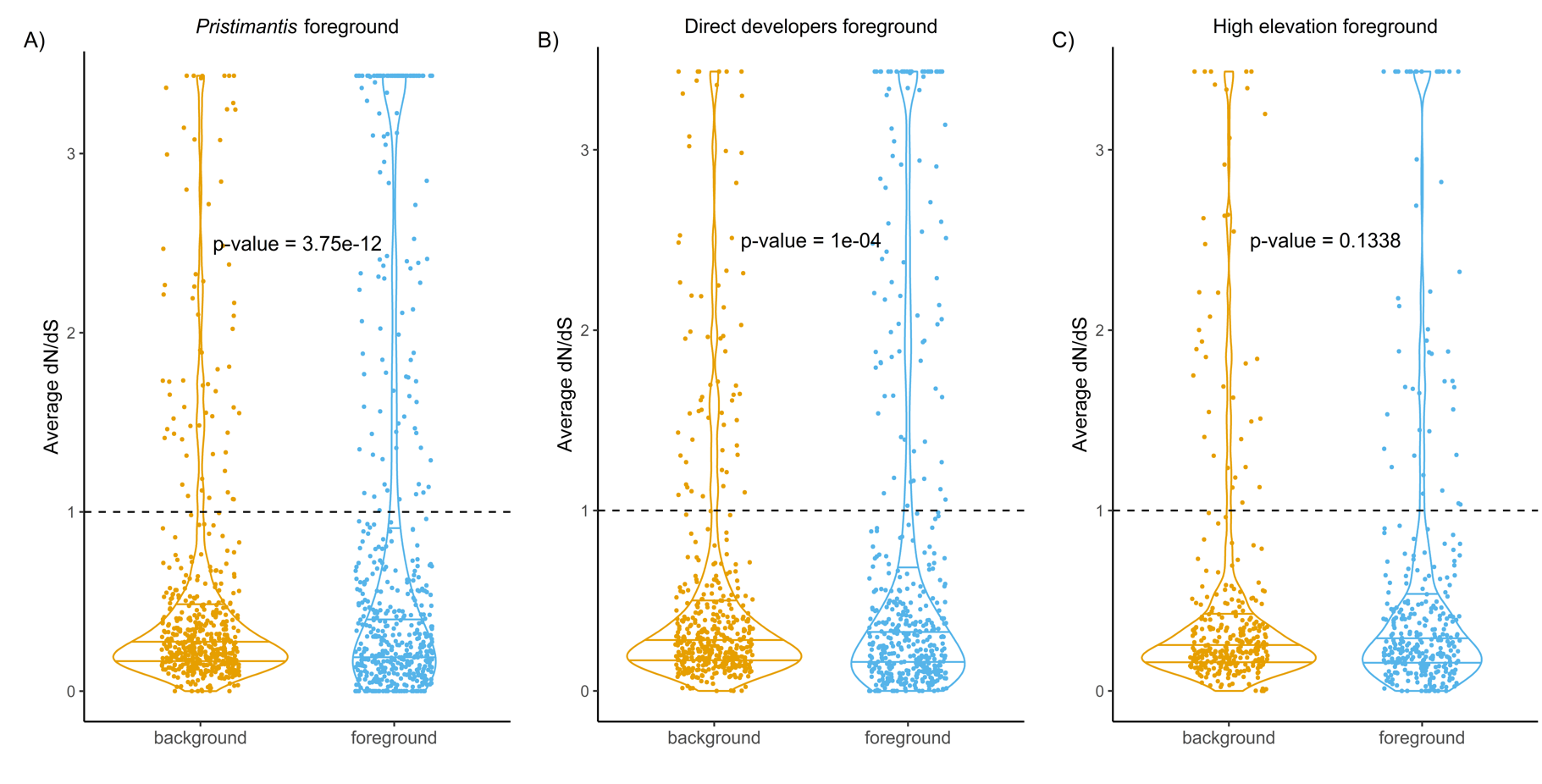
**Supplementary Figure 3.** Violin plot of average dN/dS (ω) values estimated by BUSTED across all relevant orthogroup gene trees with (A) *Pristimantis*, (B) direct developers and (C) high-elevation species set as foreground branches and all other species set as background species. P-values are from two-way paired t-tests comparing average ω values between foreground and background gene tree branches.


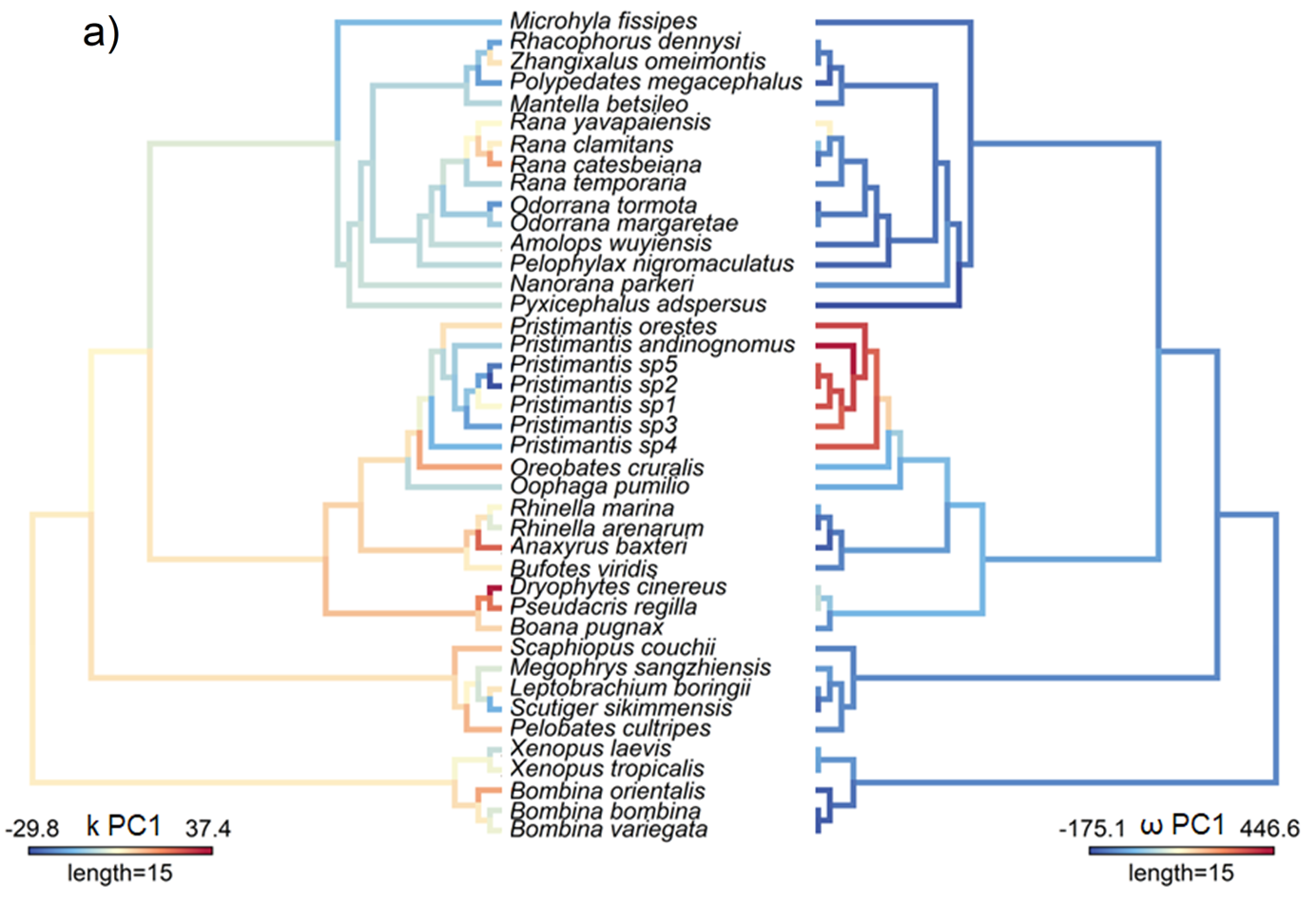


**Supplementary Figure 4.** Visualization of PC1 scores estimated by k relaxation parameter values from RELAX (left) and ω values from aBSREL (right) across the species tree with *Pristimantis* (a), direct developing (b), and high elevation (c) species only labeled as the foreground. These ω values for each orthogroup are used as inputs into a principal component analysis, and the top principal component that accounts for the highest proportion of variance between species is what is plotted along branches.


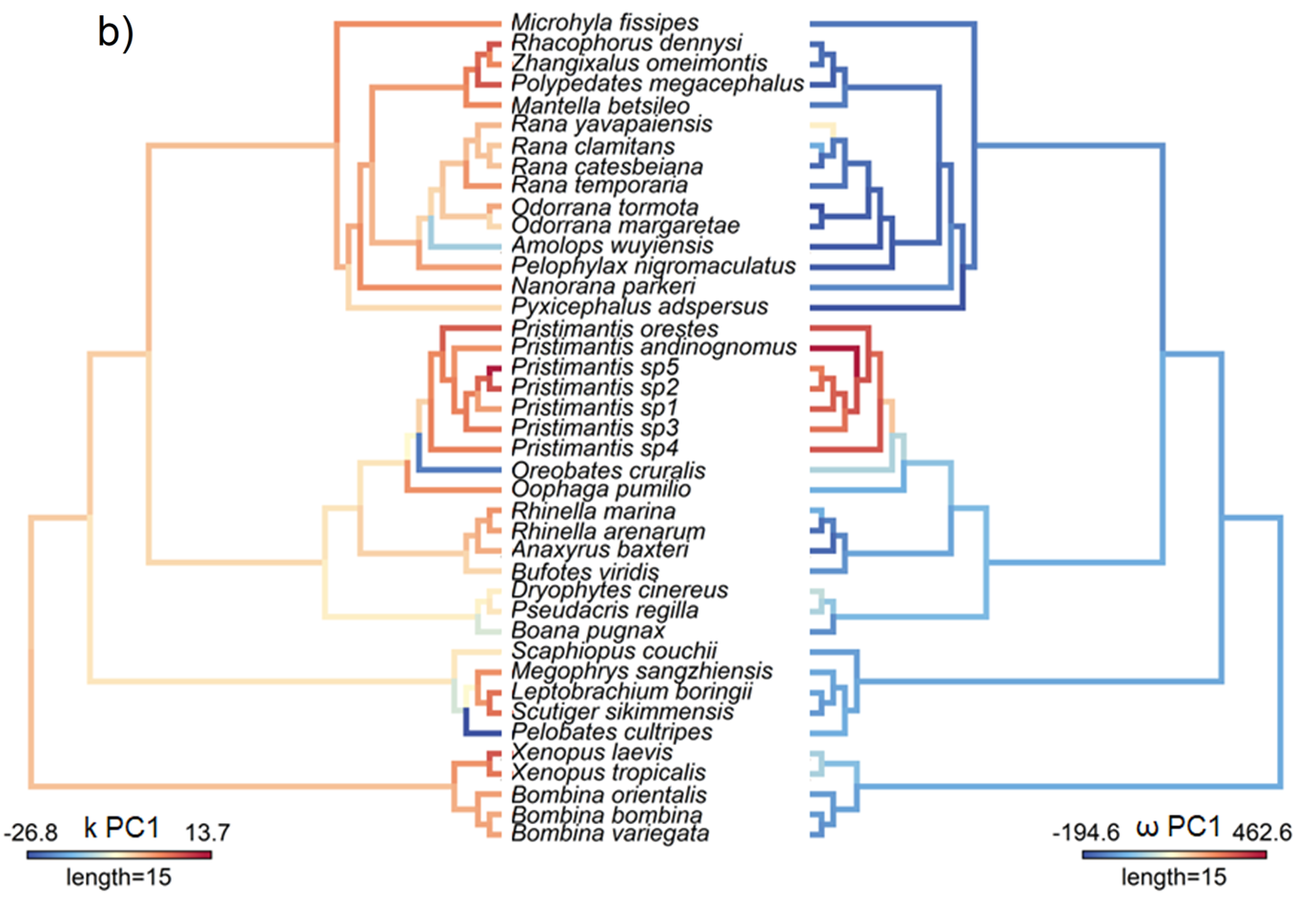


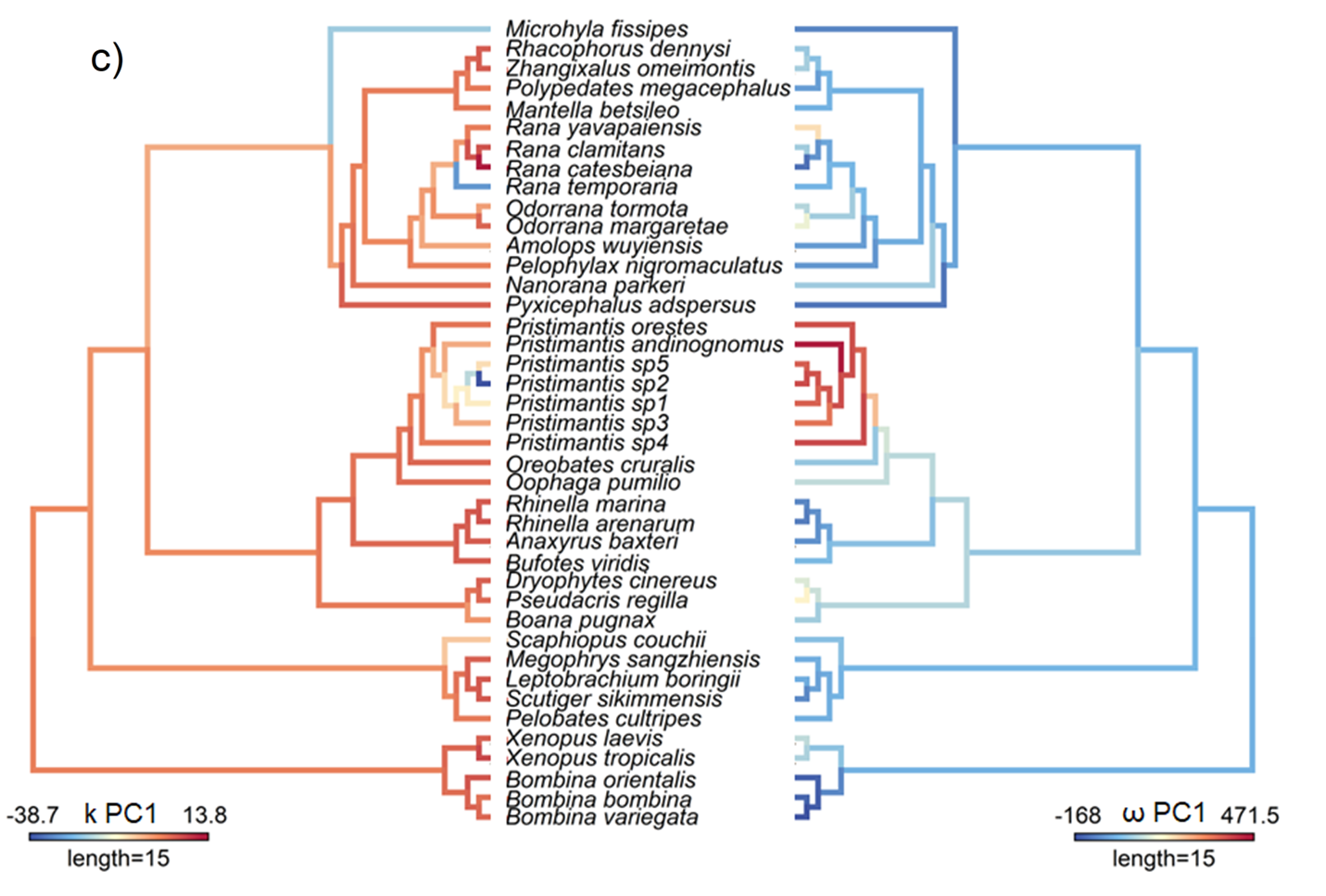


.


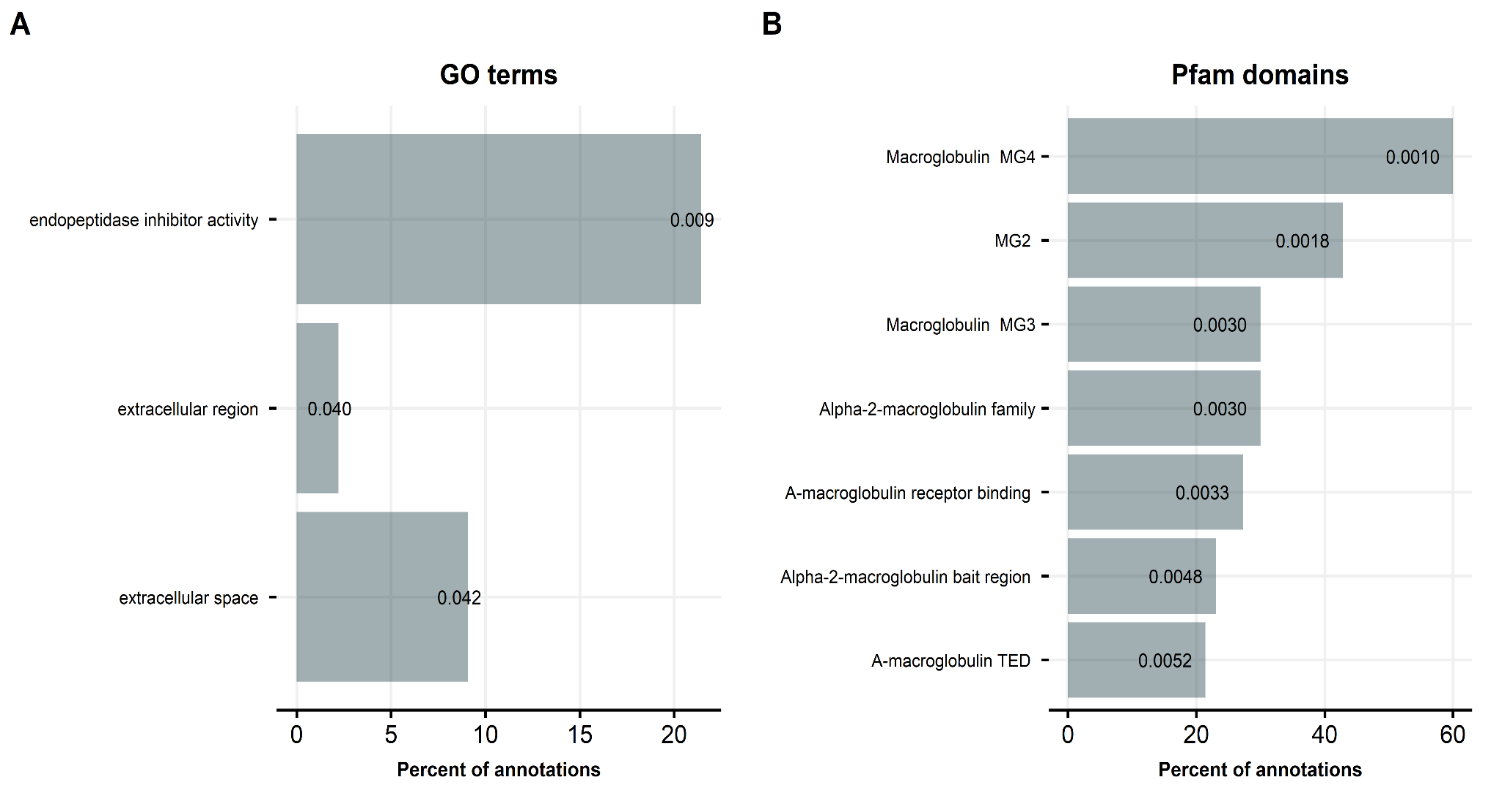


**Supplementary Figure 5.** Overrepresented (A) Gene Ontology terms and (B) Pfam domains of duplicated orthogroups (Fisher’s exact test, FDR p < 0.05). Bar size corresponds to the percentage of the total number of orthogroups annotated with Pfam or GO term from the Kinfin data files. Numbers within the bars are FDR p-values for each overrepresented category.


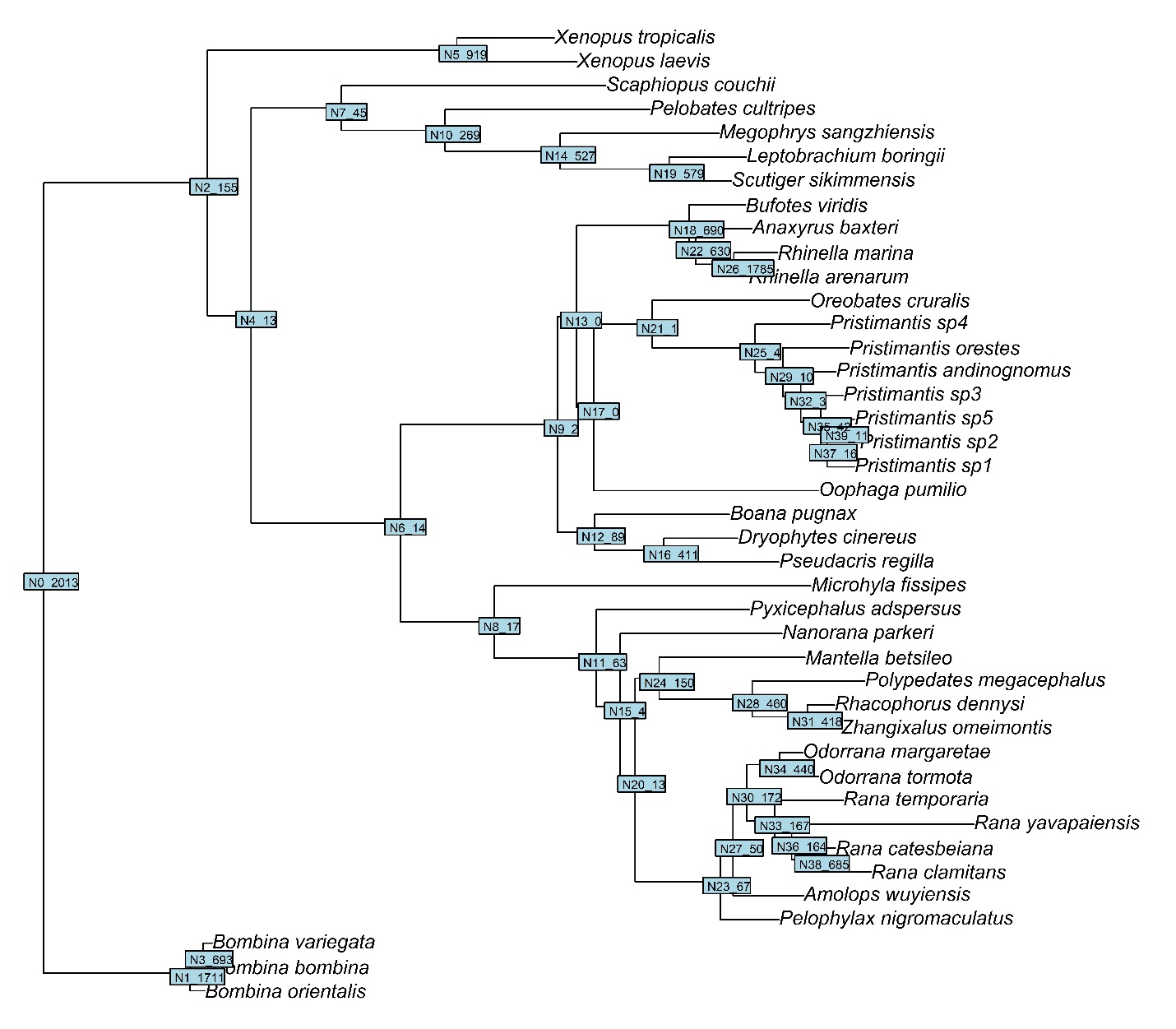


**Supplementary Figure 6.** Rooted species tree with node name (“N_”) and number of orthogroup duplications labeled at nodes. Duplicated orthogroups at nodes within the *Pristimantis* group were tested for positive selection.


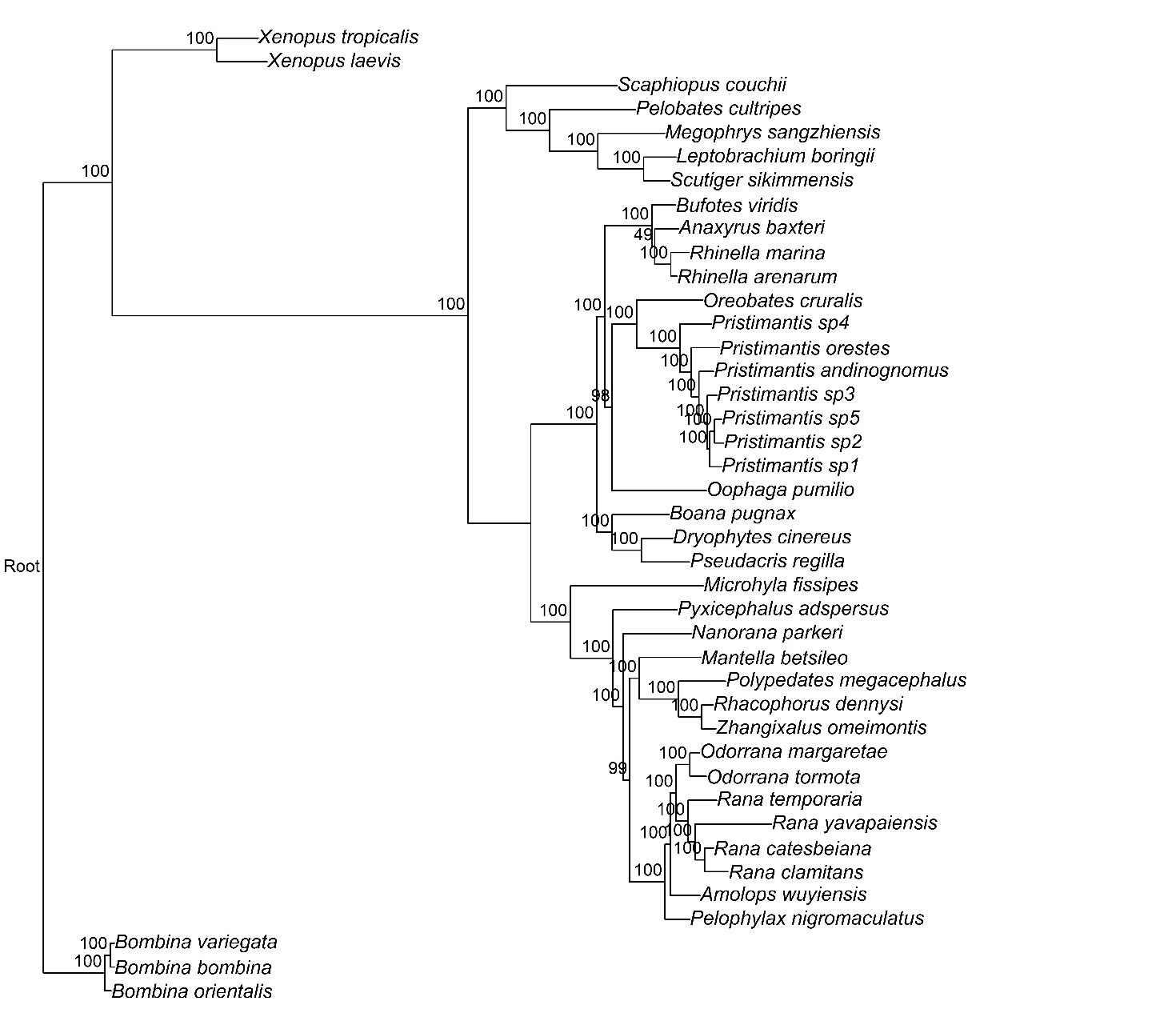


**Supplementary Figure 7.** Rooted species tree with bootstrap support values labeled on nodes.
